# CORE-Net: A Collaborative Optimization Framework for Rotated Ship Detection in Complex SAR Scenes

**DOI:** 10.3390/s26123707

**Published:** 2026-06-10

**Authors:** Yongqi Kang, Haiping Qu

**Affiliations:** School of Computer and Artificial Intelligence, Ludong University, No. 186 Hongqi Middle Road, Yantai 264025, China; kyq2826575405@163.com

**Keywords:** synthetic aperture radar (SAR), rotated ship detection, feature alignment, cascade regression, sample reliability

## Abstract

**Highlights:**

**What are the main findings?**

- This study proposes CORE-Net, a collaborative optimization framework that integrates RCFP, PCR Head, OAREU, and UARS to jointly address directional mismatch, unstable angle regression, and supervision-quality variation in complex SAR rotated ship detection.
- Ablation and comparative experiments demonstrate that the proposed method yields consistent improvements in AP_50:95_ while preserving high Recall and Precision, with gains being particularly pronounced in inshore scenes.

**What are the implications of the main findings?**

- These findings indicate that multi-level collaborative optimization constitutes an effective paradigm for enhancing both robustness and fine-grained localization quality in rotated ship detection under complex SAR conditions.
- The proposed design principles may offer instructive insights for related tasks involving rotated object detection in the presence of noisy backgrounds, arbitrary orientations, and dense target distributions.

**Abstract:**

Rotated ship detection in complex synthetic aperture radar (SAR) scenes remains a critical yet challenging task for maritime remote sensing applications. Existing methods are plagued by three core bottlenecks: inconsistent directional responses across multi-scale features, unstable rotation angle regression, and non-uniform supervision quality of positive samples during training, which collectively lead to elevated false alarms, missed detections, and severe localization degradation, especially under high IoU thresholds in complex inshore environments. To address these challenges, we propose CORE-Net, a collaborative optimization framework integrating three dedicated modules in the forward detection stage: a Rotation-Consistent Feature Pyramid (RCFP) to alleviate cross-scale directional mismatch, a Progressive Cascade Rotation Head (PCR Head) to improve progressive angle prediction stability, and an Orientation-Aware Regression Enhancement Unit (OAREU) to strengthen directional geometric representation in regression features, alongside an Uncertainty-Aware Sample Reliability Steering (UARS) module for training-stage optimization to softly downweight the regression contribution of positive samples with high classification confidence but low geometric consistency. Extensive experiments on three public SAR ship detection datasets (RSDD-SAR, SSDD+, and RSAR) demonstrate that the proposed method consistently improves AP_50:95_ while maintaining high Recall and Precision, validating that joint optimization of feature representation, rotated regression, and sample reliability is an effective strategy to enhance both the robustness and fine-grained localization capability of rotated ship detection in complex SAR scenes. In addition, large-scene inference experiments on uncropped Sentinel-1 and RSDD-SAR images further demonstrate that CORE-Net can be extended from patch-based evaluation to high-resolution SAR scene interpretation using a sliding-window inference strategy.

## 1. Introduction

Synthetic aperture radar (SAR) is an active microwave remote sensing instrument that delivers all-weather, day-and-night, wide-swath imaging. As a pivotal imaging sensor for maritime situational awareness, SAR has demonstrated exceptional value in marine surveillance, vessel traffic management, resource monitoring, and national defense security. Automatic ship detection from SAR imagery constitutes a fundamental sensing task for constructing ocean surface target perception systems. Nevertheless, ship detection in complex sensing scenes—especially inshore and harbor areas—continues to face severe challenges. Intrinsic speckle noise, combined with heterogeneous sea–land clutter and strong scattering from port facilities, degrades the discriminability of target signatures and compromises sensor image quality [1]. Furthermore, ship targets frequently present arbitrary orientations, pronounced scale variations, elongated aspect ratios, and dense distributions, which together render stable feature representation and precise localization considerably more difficult for downstream signal processing and data fusion pipelines [2]. Under such challenging sensing conditions, traditional detection methods and early deep learning models that rely on horizontal bounding boxes often produce elevated false alarm rates, missed detections, and inaccurate localization, failing to meet the demands of high-precision remote sensor interpretation [3].

To enhance the sensing capability for ship targets in complex scenes, rotated object detection has emerged as a pivotal research direction within the SAR community. Recent years have witnessed advances in rotated-box parameterization [3,4], rotation-sensitive loss functions [5,6], noise suppression and feature enhancement [7,8], and directional modeling with regression optimization [9,10,11]. While these methods have improved target representation, directional awareness, and rotated-box fitting accuracy for SAR image sensors, most of them focus on optimizing isolated components of the detection pipeline—for instance, strengthening feature alignment, introducing cascade regression, or augmenting directional sensitivity. For the multi-source error coupling inherent in complex sensor-imaging conditions, such piecewise improvements often fail to unlock the full potential of holistic sensor data fusion and processing-chain optimization.

Further analysis reveals that the performance bottleneck in sensor-based rotated ship detection does not originate from a single weak module; rather, it is intrinsically related to the coupling of errors across input interference, feature representation, rotated regression, and training supervision. Specifically, speckle noise and sea–land clutter at the sensor-imaging stage distort target representation quality. During multi-scale feature fusion, inconsistent directional responses may arise across sensor channels, complicating subsequent rotated-box modeling. Additionally, the periodicity and boundary discontinuity of the angle parameter make one-step direct regression inherently unstable for angle-sensing tasks. Moreover, the supervisory quality of positive samples varies during training, which can perturb model convergence and limit the final localization accuracy of the AI-enabled sensor system [1]. Among these issues, a critical yet often overlooked problem is the existence of positive samples that combine high classification confidence with low geometric consistency between predicted and ground-truth boxes. Such “high-confidence, low-localization-consistency” samples introduce unstable supervisory signals into the regression branch, thereby disrupting optimization and hindering localization improvements under high Intersection-over-Union (IoU) thresholds [12]. While existing training optimization strategies—including dynamic sample assignment [13] and optimal transport assignment [14]—primarily concentrate on positive/negative sample selection heuristics, they usually neglect the fine-grained modeling and regulation of supervisory quality discrepancies inside the already assigned positive sample set.

To address these limitations, this study proposes a collaborative optimization framework for rotated ship detection in complex SAR sensing scenes, termed CORE-Net. The framework jointly refines the detection process across three critical dimensions of remote sensor data processing: feature representation, regression prediction, and training regulation. The goal is to enhance both the stability and the fine-grained localization capability of rotated ship detection under complex background conditions. In the forward detection stage, the proposed architecture comprises a Rotation-Consistent Feature Pyramid (RCFP), a Progressive Cascade Rotation Head (PCR Head), and an Orientation-Aware Regression Enhancement Unit (OAREU). The RCFP performs directional consistency calibration prior to multi-scale feature fusion, mitigating directional conflicts that arise during cross-level aggregation in sensor-derived feature pyramids. The PCR Head improves the stability of rotated-parameter prediction through a coarse-to-fine, two-stage angle-modeling strategy. Concurrently, the OAREU strengthens the capacity of regression features to encode target orientation and geometric structure along the regression pathway. In the training phase, we further introduce an Uncertainty-Aware Sample Reliability Steering (UARS) module. Without altering the original sample assignment protocol, UARS leverages the observed mismatch between classification confidence and geometric consistency to softly down-weight the regression contribution of potentially unreliable positive samples. This targeted regulation reduces the disruptive influence of unstable gradients on model optimization. UARS is designed to improve the training stability and the reliability of the learned sensor model. By synergistically combining forward structural enhancement with training-stage regulation, CORE-Net is designed to more effectively counteract the performance degradation induced by multi-level error coupling in complex SAR sensor image interpretation.

The principal contributions of this study are summarized as follows:

- We propose CORE-Net, a collaborative optimization framework for rotated ship detection in complex SAR sensor scenes. It unifies multi-scale directional consistency modeling, progressive rotated regression, and training-stage sample reliability regulation within a cohesive pipeline to jointly improve detection stability and localization accuracy in challenging sensing environments.
- We design three complementary modules: a Rotation-Consistent Feature Pyramid (RCFP) for directional calibration prior to feature fusion, a Progressive Cascade Rotation Head (PCR Head) for progressive angle prediction, and an Orientation-Aware Regression Enhancement Unit (OAREU) for augmenting directional information in regression features. These modules are tailored to enhance the geometric perception capability of the AI-enabled sensor system.
- We introduce an Uncertainty-Aware Sample Reliability Steering (UARS) module. Operating online without altering the base sample assignment, UARS identifies supervisory quality variations among positive samples and applies a soft down-weighting exclusively to the regression branch of unreliable samples, thereby enhancing training stability and the reliability of the learned sensor model.
- Extensive experiments on public SAR ship detection benchmarks, including RSDD-SAR, SSDD+, and RSAR, demonstrate that the proposed method improves rotated-box localization under high-IoU thresholds while maintaining robust detection capability. In addition, qualitative large-scene inference on uncropped Sentinel-1 and RSDD-SAR images verifies the scalability of CORE-Net from patch-based evaluation to high-resolution SAR scene interpretation.

## 2. Related Work

Rotated ship detection in complex SAR scenes encompasses multiple interconnected research areas, including multi-scale feature representation, rotated box regression, and training sample optimization. To contextualize the proposed methodology, this section reviews the relevant literature across four key perspectives: feature alignment and directional modeling in rotated object detection, cascade optimization for rotated box regression, rotated ship detection methods in SAR imagery, and sample quality-aware optimization strategies during training. This analysis underscores the critical need for multi-scale directional consistency modeling, progressive angle regression, and positive-sample reliability regulation in complex scenes.

### 2.1. Feature Alignment and Orientation Modeling in Rotated Object Detection

A fundamental challenge in rotated object detection lies in the geometric mismatch induced by arbitrary object orientations. Conventional convolutional features extract local responses based on fixed-shaped receptive fields, which struggle to reliably capture discriminative information distributed along an object’s principal axis. To mitigate this, researchers have developed various feature alignment and directional modeling techniques, broadly categorized as explicit spatial transformations or implicit feature enhancements.

Early approaches predominantly employed explicit spatial transformations to achieve directional alignment. For instance, SCRDet [15] utilizes a sampling alignment module with deformable convolution [16] to adaptively adjust sampling locations, enabling convolutional kernels to align along the target’s orientation. The RoI Transformer [17] maps horizontal region proposals into rotated proposals via a dedicated learner and performs region-level feature alignment. Oriented RepPoints [18] represents objects as adaptively learned point sets and employs directional transformation functions to flexibly model the geometric structure of arbitrarily oriented targets, transitioning from box regression to point-set representation.

In multi-scale feature pyramid structures, directional modeling becomes increasingly intricate. Deep features possess strong semantic representation but low spatial resolution, with directional perception biased toward global contours. Conversely, shallow features offer rich edge- and texture-related details, but are more susceptible to noise and local disturbances. Direct summation or concatenation of features with divergent directional preferences often introduces conflicting information into the fused output, complicating rotated parameter modeling for subsequent detection heads. To alleviate this, attention mechanisms have been integrated post-fusion. For example, FGAA-FPN [19] introduces angle-aware multi-head attention to enhance semantic interactions among high-level features by modeling relative directional relationships. FAFNet [20] notes that direct element-wise addition in conventional FPNs can introduce misaligned features, and proposes a Feature Selection and Alignment Pyramid Network (FSA-FPN). CGA-Det [11] enhances the rotational discriminability of detection-head inputs using direction-sensitive convolution.

More recently, researchers have begun explicitly addressing cross-level directional consistency during the fusion process. FAAFusion [21] leverages the rotational equivariance of the Fourier transform in the frequency domain to calibrate high-level feature orientation to the principal direction of low-level features prior to FPN fusion, thereby achieving cross-scale directional consistency modeling. ReAFFPN [22] strengthens inter-level feature fusion via rotation-equivariant attention, mitigating bottlenecks caused by semantic discontinuities and scale differences. These studies collectively indicate that explicitly modeling directional consistency within feature pyramids is beneficial for improving rotated object detection performance.

In summary, existing feature alignment methods are predominantly applied post-fusion or are confined to directional enhancement within individual feature levels. The modeling of cross-level directional consistency prior to fusion remains underexplored. Particularly in complex SAR scenes, directional conflicts between shallow textures and deep semantics can significantly impede subsequent rotated parameter modeling. Therefore, implementing directional consistency calibration before feature fusion represents a promising avenue for further investigation.

### 2.2. Cascade Optimization and Angle Refinement for Rotated Box Regression

Another key challenge in rotated object detection lies in the periodicity and boundary discontinuity inherent in angle parameters. For elongated SAR ship targets with large aspect ratios in SAR images, directly regressing single-stage angle regression is prone to prediction oscillation and localization inaccuracies, thereby degrading high-precision detection. To address this, coarse-to-fine cascade regression strategies have been introduced to iteratively refine angle predictions and improve localization fidelity.

In generic object detection, Cascade R-CNN [23] established a foundational paradigm for cascade regression by iteratively refining bounding boxes with detection heads operating at progressively increasing IoU thresholds. This concept has been successfully adapted for rotated detection. Rotated Cascade R-CNN [24] extends the cascade structure to rotated boxes, progressively calibrating proposals via a rotated cascade region proposal network. S^2^A-Net [25] integrates feature alignment with angle refinement to achieve multi-step rotated box refinement within a single-stage framework. Methods such as Gliding Vertex [26] and Oriented R-CNN [27] similarly embody coarse-to-fine principles via strategies of “coarse horizontal localization + independent angle refinement.”

Single-stage detectors are often preferred for practical SAR image analysis due to their inference speed and deployment convenience. However, realizing lightweight progressive angle modeling within a single-stage architecture remains non-trivial. R^3^Det [28] represents a notable single-stage rotated refinement detector that achieves higher precision through multi-stage feature iteration. Nonetheless, interactions between stages are typically limited to simple feature re-encoding, with underutilized angle priors, and the additional multi-stage processing introduces computational overhead. In contrast, general real-time detectors like RTMDet [29] rely primarily on one-step angle prediction when adapted for rotated tasks, lacking an explicit cascade refinement mechanism.

In general, existing cascade regression methods confirm that coarse-to-fine rotated box modeling is effective for mitigating angle prediction instability. Yet, most approaches remain tied to two-stage frameworks or multi-stage serial refinement structures. Lightweight progressive angle regression integrated within a single-stage detection head warrants further investigation. Furthermore, the utilization of angle priors from preceding stages remains rudimentary, leaving room for improved cross-stage transmission of directional information.

### 2.3. Challenges and Methods for Rotated Ship Detection in SAR Images

SAR imagery presents unique challenges, including multiplicative speckle noise, strong sea–land clutter, complex port scattering, and dense target distributions in inshore backgrounds. Ship targets in SAR images often exhibit elongated shapes, arbitrary orientations, large-scale variations, and weak boundaries, which make robust target discrimination and accurate rotated bounding-box localization difficult.

Traditional SAR ship detection methods, such as CFAR-based detectors and handcrafted feature-based approaches, have been widely investigated [30,31]. These methods usually exploit local clutter statistics, backscattering intensity, or SAR-specific physical descriptors to distinguish ships from sea clutter. For example, VV/VH responses, dual-polarization correlation information, and SPAN-like intensity representations can provide physically interpretable cues for ship detection [32]. However, their performance is inherently limited by rigid clutter distribution assumptions, manual threshold tuning, dependence on polarization data availability, and vulnerability to complex inshore structures [31]. In heterogeneous nearshore scenes, strong scattering from ports, coastlines, islands, and man-made facilities frequently causes severe false alarms and missed detections [33].

With the development of deep learning, learning-based SAR ship detection methods have shown stronger adaptability to complex backgrounds. Recent studies have improved SAR ship detection through rotated box representation, feature enhancement, multi-scale fusion, and direction-aware modeling. Compared with horizontal bounding boxes, rotated bounding boxes are more suitable for elongated ships with arbitrary orientations and can significantly reduce redundant background regions in dense or cluttered scenes.

In terms of direction-aware modeling, existing methods have mainly enhanced directional sensitivity by refining feature extractors or detection heads. For example, CGA-Det [11] incorporates a spatial channel-aware mechanism into the detection head to improve directional discrimination, while SARFA-Net [9] enhances rotated box regression through shape-aware label assignment and refined feature alignment. These methods demonstrate the importance of directional modeling for SAR rotated ship detection. However, most existing works still focus on local feature enhancement or the detection head itself, while the directional inconsistency among multi-scale features before feature fusion remains insufficiently explored, and they generally neglect the joint optimization of multi-scale directional consistency, progressive angle regression, and positive sample reliability.

To overcome the limitations of traditional methods, recent studies have explored integrating SAR-specific auxiliary priors and handcrafted feature guidance into deep learning frameworks. For example, SLA-Net introduces sea–land prior information and a hierarchical attention mechanism to guide the network to progressively focus on sea and ship regions, thereby reducing land-induced false alarms in inshore scenes [34]. In addition, the Laplace & LBP feature-guided SAR ship detection method incorporates edge and local texture cues into the detection network through an adaptive feature enhancement block, which improves target representation under cluttered SAR backgrounds [35]. These studies demonstrate that SAR-specific prior knowledge and local feature guidance can enhance ship discrimination and clutter suppression.

However, these prior-guided and handcrafted feature-guided methods primarily focus on background interference suppression or local target response enhancement. Moreover, sea–land prior-guided methods rely heavily on additional segmentation information, while handcrafted feature-guided methods lack the ability to model rotation-consistent multi-scale representations. Most importantly, they fail to explicitly address three critical challenges in rotated ship detection: (1) directional inconsistency among multi-scale features before fusion, (2) instability of one-step or weakly refined rotated angle regression, and (3) supervision quality variation in assigned positive samples during training.

To tackle these issues, CORE-Net proposes a collaborative optimization framework for the entire rotated detection pipeline. Specifically, the Rotation-Consistent Feature Pyramid (RCFP) calibrates multi-scale directional responses prior to feature fusion; the Progressive Cascade Rotation Head (PCR Head) refines rotated angle predictions in a coarse-to-fine manner; the Orientation-Aware Regression Enhancement Unit (OAREU) enhances orientation-aware geometric representation in the regression branch; and the Uncertainty-Aware Sample Reliability Steering (UARS) regulates positive sample quality during training. This comprehensive design enables more accurate fine-grained rotated ship localization in complex SAR scenes.

It should be emphasized that this work focuses on learning-based rotated ship detection under standard image-domain benchmark settings. We do not aim to design or systematically compare with traditional CFAR-based or physical feature-based SAR detectors. Instead, we consider CFAR algorithms, polarimetric descriptors, dual-polarization correlation features, and SPAN-like representations as important complementary techniques that can be integrated into our framework in future work.

### 2.4. Sample-Quality Awareness and Optimization During Training

Effective utilization of training samples is paramount for optimizing detection model performance. Existing research on sample quality modeling and training optimization has focused on hard sample mining, dynamic label assignment, classification–localization consistency modeling, and uncertainty estimation. However, the predominant focus has been on determining which samples should be selected as positive, with relatively limited attention paid to how supervisory quality differences should be managed after positive assignment.

In hard sample mining, OHEM [36] prioritizes high-loss samples in each training batch for gradient back-propagation, intensifying learning on challenging instances. While effective, this strategy can be aggressive and potentially introduces noise in complex scenes. Focal Loss [37] elegantly addresses class imbalance by down-weighting the loss contribution of easy-to-classify samples via a modulating factor. Nevertheless, these methods primarily define difficulty based on classification performance, lacking specificity for the more nuanced geometric regression challenges inherent in rotated detection tasks.

In dynamic label assignment, conventional fixed IoU threshold strategies often struggle to accommodate variations in target scale and scene complexity. ATSS [38] adaptively determines the positive/negative threshold based on the statistical distribution of candidate box IoUs. OTA [39] formulates label assignment as a global optimal transport problem and demonstrates strong adaptability in dense target scenarios. Such methods significantly enhance the quality of positive/negative partitioning. Yet, once assignment is finalized, all positive samples are typically treated uniformly during training, disregarding the heterogeneous supervisory quality within the positive set.

In classification–localization consistency modeling, studies have observed that classification confidence can be incongruent with localization accuracy and have sought to rectify this via quality modeling. IoUNet [40] predicts the IoU between the predicted box and the ground-truth box as a proxy for localization quality, which can be utilized for non-maximum suppression or loss weighting. FCOS [41] introduces a “centerness” branch to quantify the degree of center deviation. Generalized Focal Loss (GFL) [42] jointly models classification scores and localization quality. These methods, however, are primarily designed for confidence calibration during inference or weight adjustment during label assignment; the soft regulation of regression supervision for already assigned positive samples during training receives comparatively less attention.

In uncertainty estimation, Bayesian deep learning techniques estimate predictive variance using methods like Monte Carlo Dropout [43] or model ensembling. This uncertainty can inform loss weighting or reliable prediction selection. However, such methods typically incur high computational cost, and the estimated uncertainty reflects global statistical uncertainty rather than the instantaneous reliability of a specific sample’s supervisory signal at a given training iteration.

In summary, existing works have advanced training processes through hard sample mining, dynamic assignment, quality calibration, and uncertainty modeling. Yet, the majority concentrate on sample selection or confidence modeling, with insufficient exploitation of supervisory quality variations within the assigned positive sample set. Notably, positive samples with high classification responses but low geometric consistency can inject instability into the regression branch during training. Consequently, finer-grained training regulation predicated on reliability differences among positive samples merits deeper investigation.

Overall, prior studies have enhanced rotated object detection performance from the standpoints of feature alignment, rotated regression, SAR scene modeling, and training optimization, but most methods still focus on improving only a single stage of the pipeline. For rotated ship detection in complex SAR scenes, challenges such as multi-scale directional mismatch, unstable angle regression, and fluctuations in positive-sample supervision quality often coexist and interact during training. Therefore, a joint optimization strategy that holistically addresses feature representation, regression prediction, and training regulation holds considerable promise for further improving detection stability and fine-grained localization accuracy in complex environments.

## 3. CORE-Net Method

### 3.1. Overall Architecture

**Figure 1:**
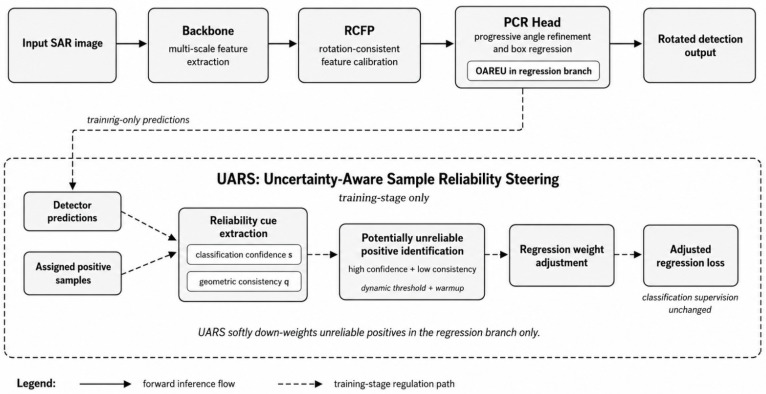
Overall architecture of CORE-Net. The framework consists of a forward detection pipeline and a training-stage reliability regulation mechanism. The forward pipeline integrates the Backbone, RCFP, PCR Head, and OAREU to enhance rotation-consistent feature representation and rotated box prediction, while UARS is activated only during training to adjust regression weights for potentially unreliable positive samples.

To simultaneously enhance the stability and localization accuracy of rotated ship detection in complex SAR scenes, we propose CORE-Net, a collaborative optimization framework whose overall architecture is conceptually illustrated in Figure 1. The framework comprises two core components: a forward detection pipeline and a training-stage sample reliability regulation mechanism. The forward detection pipeline consists of a feature extraction backbone, a Rotation-Consistent Feature Pyramid (RCFP), a Progressive Cascade Rotation Head (PCR Head), and an Orientation-Aware Regression Enhancement Unit (OAREU). During training, an Uncertainty-Aware Sample Reliability Steering (UARS) module is additionally introduced to stabilize regression optimization.

An input SAR image is first processed by the backbone to extract initial multi-scale features. The RCFP then calibrates the directional responses across different feature levels, mitigating cross-level directional conflicts that would otherwise degrade subsequent fusion. The aligned multi-scale features are forwarded to the detection head, where the PCR Head progressively models target position, scale, and orientation, while the OAREU augments directional geometric cues within the regression branch, thereby strengthening both the stability and discriminability of rotated-box learning. Final predictions are decoded and post-processed to yield rotated bounding boxes.

Notably, UARS is excluded from forward inference. Instead, after positive-sample assignment, it evaluates the supervision quality of positive samples on the fly based on classification confidence and geometric consistency. Positive samples that exhibit high classification confidence yet low geometric consistency are softly down-weighted exclusively in the regression branch, while classification supervision remains unaffected.

In summary, the RCFP enforces directional consistency across multi-scale features, the PCR Head improves the stability of progressive angle prediction, the OAREU enhances directional geometric representation in regression features, and the UARS module regulates positive sample reliability during training. These four modules operate at complementary stages of the detection pipeline and collectively advance rotated ship detection in challenging SAR environments.

### 3.2. Rotation-Consistent Feature Pyramid (RCFP)

In rotated object detection, target orientation constitutes a critical cue for inferring the geometric parameters of rotated bounding boxes, most notably the angle. However, in feature-pyramid-based multi-scale detection frameworks, the directional responses elicited by the same rotated target often vary inconsistently across feature levels. Deep features possess stronger semantic representational capacity, yet their coarse spatial resolution biases orientation perception toward global contours. Conversely, shallow features retain richer edge- and texture-related details but are more susceptible to speckle noise, sea–land clutter, and local scattering interference. When features with disparate directional preferences are naively fused via addition or concatenation in the FPN/PAN, the resulting representations tend to embed conflicting directional information. This complicates the joint modeling of target center, scale, and angle in the subsequent detection head, often manifesting as unstable angle estimation, orientation jitter in predicted boxes, and degraded localization performance under stringent IoU thresholds. Fundamentally, this issue stems from a directional representation mismatch across feature levels prior to fusion, and it is particularly pronounced in scenarios involving elongated ships, complex Inshore backgrounds, and partial occlusion.

To mitigate this issue, we propose a Rotation-Consistent Feature Pyramid (RCFP), which calibrates the directional responses of multi-scale features prior to their input into subsequent fusion layers and the detection head. Unlike explicit geometric rotation methods that rely on affine transformation or spatial resampling, the RCFP does not directly modify the spatial coordinates of feature maps. Instead, it performs soft directional alignment in the feature domain via three core operations: multi-directional response modeling, direction-aware weight allocation, and gated residual fusion. This design preserves the continuity of end-to-end training, while avoiding the additional computational overhead and quantization errors introduced by explicit rotational interpolation.

**Figure 2:**
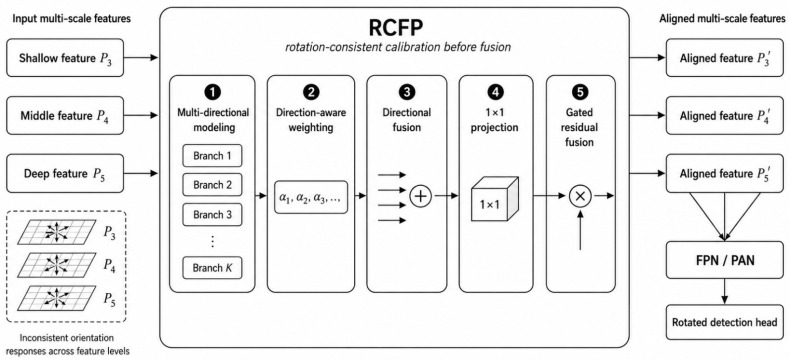
Structure of the Rotation-Consistent Feature Pyramid (RCFP). RCFP calibrates inconsistent directional responses at each feature level and produces aligned multi-scale features before FPN/PAN fusion.

As illustrated in Figure 2, an identical directional consistency calibration workflow is applied to shallow, intermediate, and deep features. For an input feature map X at any level, multiple direction-sensitive branches are first employed for parallel expansion to generate a set of directional features. Each branch corresponds to a unique directional response pattern, and is responsible for capturing responses associated with the target’s principal axis, edge contour, and local texture from a specific orientation. This process can be formulated as

(1) $$D_{k}=\varphi_{k}(X),k=1,2,\ldots ,K$$

where $\varphi_{k}(\cdot )$ denotes the feature mapping function of the *k*-th directional branch. Through this parallel directional expansion process, the original directional information entangled in the channels is explicitly decomposed into multiple candidate directional components, providing a foundational basis for subsequent direction selection and consistency modeling.

After obtaining the multi-directional responses, RCFP further introduces a lightweight direction-weight estimation module to adaptively model the importance of each directional branch. Specifically, a score is first generated for each direction based on the input feature or the multi-directional features, and then normalized to yield the corresponding directional weight $\alpha_{k}$. This process can be written as

(2) $$\alpha_{k}=\frac{\exp(s_{k})}{\sum_{j=1}^{K}\exp(s_{j})}$$

where $s_{k}$ represents the response score of the *k*-th directional branch, and $\alpha_{k}$ quantifies the relevance of that directional component to the current sample. Leveraging these weights, the multi-directional features are fused into a direction-aligned representation. Critically, this mechanism enables the network to adaptively amplify directional components that align with the target’s dominant orientation while suppressing those heavily corrupted by background interference or inconsistent with the primary direction. In this manner, cross-level directional discrepancies are mitigated prior to feature fusion.

Nevertheless, relying exclusively on directional aggregation risks perturbing the original feature distribution. Hence, following directional fusion, RCFP further introduces a 1 × 1 projection layer and a gated residual branch. First, a 1 × 1 convolution projects and compresses the direction-fused feature into a compact representation. Subsequently, the projected feature Z and the original feature X are jointly fed into a gating unit to generate a gating coefficient G, after which residual reconstruction is performed. This process is formulated as

(3) $$G=\sigma (\gamma (\left[Z,X\right]))$$

(4) Y=G⊙Z+(1−G)⊙X

where γ(⋅) denotes the gating mapping function, σ(⋅) is the Sigmoid function, and ⊙ represents element-wise multiplication. By virtue of this design, RCFP does not merely substitute the original feature with its direction-aligned counterpart. Instead, it dynamically strikes a balance between direction-enhanced information and the original response. When the directionally calibrated information proves more reliable, the gated branch increases its contribution; when the original feature is already sufficiently stable, the module retains a larger proportion of the original response. This residual fusion strategy promotes training stability while bolstering directional consistency.

Following the aforementioned processing, the input features P3, P4, and P5 are mapped into direction-calibrated aligned features P3′, P4′, and P5′, respectively. These features are no longer mere multi-scale semantic representations; rather, they constitute detection-head inputs with substantially more consistent directional responses. The aligned multi-scale features are then passed to the subsequent FPN/PAN for further fusion and ultimately fed into the rotated detection head for bounding box prediction. Because cross-level features have already undergone preliminary directional coordination before reaching the detection head, the regression branch can devote greater capacity to fine-grained modeling of target center, scale, and angle, without additionally contending with interference arising from pre-fusion directional conflicts.

### 3.3. Progressive Cascade Rotation Head (PCR Head)

In conventional single-stage rotated object detectors, the target center, width, height, and rotation angle are typically regressed simultaneously by a unified detection head in a single forward pass. Although this “one-shot” prediction paradigm affords high inference efficiency, it often exhibits considerable instability when handling angle parameters that are inherently periodic and discontinuous at boundaries. For ship targets in complex SAR scenes, local features are readily corrupted by strong scattering points, speckle noise, intricate shoreline structures, and partial occlusion, rendering angle prediction overly sensitive to instantaneous feature quality. Once the initial angle estimate deviates from the true orientation, the error propagates directly into the rotated bounding box decoding process, thereby exacerbating inaccuracies in both directional estimation and localization. Consequently, replacing one-step direct angle regression with a stage-wise progressive prediction strategy constitutes an effective means of enhancing the stability of rotated box regression.

**Figure 3:**
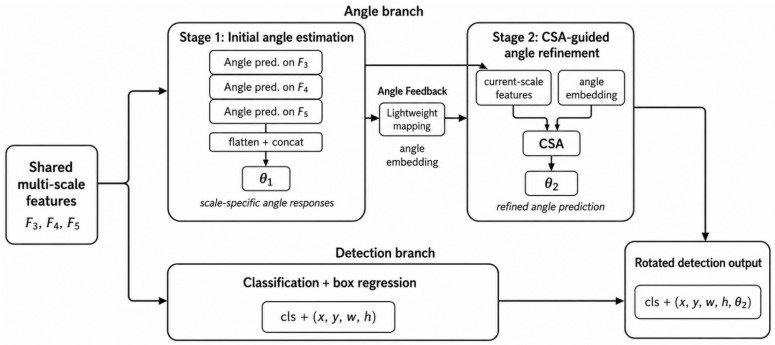
Structure of the Progressive Cascade Rotation Head (PCR Head). The angle branch progressively refines the rotation angle from θ_1_ to θ_2_ using angle embedding feedback and CSA, while the detection branch predicts the category and geometric parameters (x, y, w, h).

Motivated by this insight, we design a Progressive Cascade Rotation Head (PCR Head), whose overall architecture is depicted in Figure 3. In contrast to standard detection heads that output (x, y, w, h, θ) in a unified fashion, the PCR Head adopts a relatively decoupled design for the detection and angle branches. Specifically, the detection branch is responsible for category prediction and regression of the geometric parameters (x, y, w, h), whereas the angle branch is dedicated to a two-stage cascaded prediction of the rotation angle. In implementation, the PCR Head operates on the multi-scale features F3, F4, and F5—corresponding to detection strides of 8, 16, and 32, respectively—that are fed into the detection head. Both stages share the same set of multi-scale input features, obviating the need to re-extract region features at each stage as in conventional multi-stage frameworks. This design maintains a streamlined detection pipeline while enabling progressive refinement of the angle parameter.

As illustrated in Figure 3, the angle branch comprises two sequential stages. The first stage generates an initial angle estimate. For the input features F3, F4, and F5 at different scales, each is first passed through its corresponding angle prediction sub-branch to obtain scale-specific angle responses. These responses are then flattened along the spatial dimension and concatenated to produce the initial angle prediction θ_1_ of the first stage. The primary purpose of this stage is to provide a relatively stable estimate of the dominant orientation based on the current multi-scale features, thereby furnishing a directional prior for subsequent angle refinement. Concurrently, the angle responses generated at each scale in the first stage are further transformed by a lightweight mapping module to form corresponding angle embedding features, which are transmitted to the second stage via the Angle Feedback path shown in Figure 3. Thus, what the second stage receives is not the raw angle value predicted by the first stage, but rather a directional embedding representation derived from the preceding stage’s angle responses.

**Figure 4:**
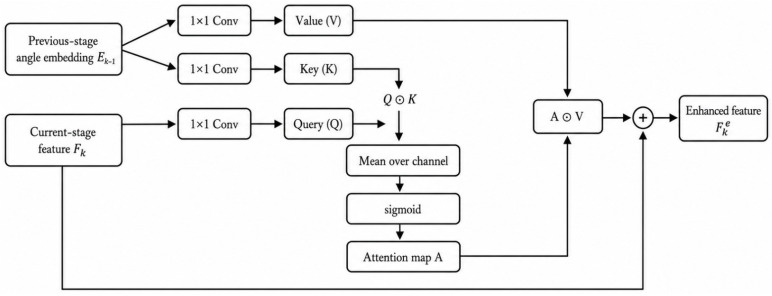
Structure of the Cross-Stage Attention (CSA) module. CSA transfers directional priors from the previous-stage angle embedding to the current-stage feature by generating an attention map from query–key interaction and enhancing the feature through value modulation and residual fusion.

Armed with the directional prior from the first stage, the second stage further refines the angle prediction. Rather than performing another independent angle prediction from scratch, the second stage incorporates the angle embedding features generated in the previous stage alongside the current-scale features, and accomplishes directional information transfer and feature enhancement through the Cross-Stage Attention (CSA) module, whose structure is shown in Figure 4.

Let the current-stage feature $F_{k}$ and the previous-stage angle embedding $E_{k-1}$ be denoted as corresponding variables. They are first projected by 1 × 1 convolutions to obtain the query, key, and value terms:

(5) $$Q=\varphi_{q}(F_{k}),\;\;\;\;K=\varphi_{k}(E_{k-1}),\;\;\;\;V=\varphi_{v}(E_{k-1})$$

where the corresponding symbols $\varphi_{q}$, $\varphi_{k}$, and $\varphi_{v}$ denote the respective convolution mapping functions.

Then, the element-wise interaction between *Q* and *K* is used to characterize the correlation between the current-stage feature and the directional prior from the previous stage. After aggregation along the channel dimension, a single-channel spatial response map is obtained:

(6) $$A=\sigma (Meam_{c}(Q⊙K))$$

where the symbol ⊙ denotes element-wise multiplication, $Meam_{c}$ channel-wise mean operation, and σ(⋅) the Sigmoid activation function, respectively.

Furthermore, the attention map *A* is employed to modulate the value branch *V*, and a learnable fusion coefficient *γ* is introduced to inject cross-stage information into the current-stage feature in a residual manner, yielding the enhanced stage feature:

(7) $$F_{k}^{e}=F_{k}+\gamma (A⊙V)$$

where *γ* is a learnable fusion coefficient used to control the strength of injecting the directional information from the previous stage into the current-stage feature. Through this design, the CSA module establishes explicit correlation modeling between the current-stage feature and the angle prior from the previous stage, thereby enhancing the utilization of directional information in the second stage.

Upon completion of cross-stage feature enhancement, the branches at different scales in the second stage output refined angle predictions, which are subsequently fused to obtain the final angle prediction θ_2_. Hence, θ_1_ and θ_2_ correspond, respectively, to the initial angle estimation and the refined angle prediction, forming a coarse-to-fine progressive relationship in functionality. Notably, the second stage does not explicitly learn the numerical residual of θ_1_; instead, it outputs a more stable final angle prediction under the guidance of the directional prior supplied by the first stage. Compared with single-stage direct angle prediction, this design helps alleviate the instability of angle modeling under complex background conditions.

In addition to the angle branch, the PCR Head also retains a parallel detection branch for predicting target categories as well as center location and scale parameters. During training, the (x, y, w, h) predicted by the detection branch and the final θ_2_ generated by the angle branch are jointly used to construct the supervision signal for rotated bounding boxes. During inference, the predicted geometric parameters and the final angle prediction are jointly decoded to produce the rotated detection results. In this manner, the PCR Head establishes a clear functional division: the detection branch handles position and scale modeling, while the angle branch manages coarse-to-fine directional modeling, thereby enhancing the stability of angle regression and localization accuracy without substantially increasing structural complexity.

Furthermore, a defining characteristic of the PCR Head is feature sharing. Unlike multi-stage detection frameworks in which region features are re-extracted at each stage, the two angle prediction stages in our design share the same set of multi-scale input features. Directional modulation and feature enhancement in the second stage are achieved solely through the angle embedding feedback from the previous stage and the CSA module. This design avoids the cumbersome process of repeated feature extraction and enables angle refinement with only a marginal increase in computational cost.

### 3.4. Orientation-Aware Regression Enhancement Unit (OAREU)

Although the RCFP mitigates cross-scale directional inconsistency at the feature pyramid level and the PCR Head reduces the sensitivity of angle prediction to one-shot estimation via progressive regression, the representational fidelity of direction-related geometric cues within the regression branch remains a decisive factor in the ultimate localization accuracy of rotated bounding boxes. For ship targets in complex SAR scenes, their geometric profiles typically manifest as elongated structures and are prone to disturbance by speckle noise, sea–land clutter, and localized strong scattering. Under such conditions, if the regression branch continues to rely predominantly on conventional convolutional features to learn bounding box parameters, several issues may ensue: insufficient directional representation, stronger entanglement between scale and angle, and amplification of local perturbations in geometric regression. These shortcomings ultimately constrain localization performance under stringent high-IoU thresholds.

**Figure 5:**
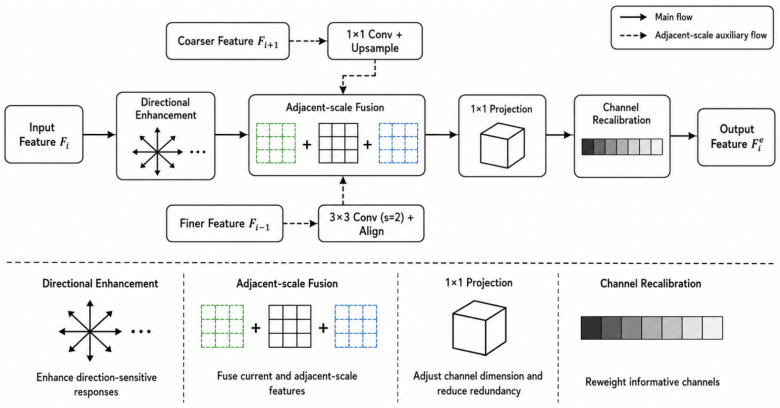
Structure of the Orientation-Aware Regression Enhancement Unit (OAREU). The module enhances the current-scale regression feature via directional enhancement, adjacent-scale auxiliary fusion, 1 × 1 projection, and channel recalibration, thereby generating a more stable and geometrically discriminative feature representation for rotated box regression. Different colors are used to distinguish the current-scale feature and adjacent-scale auxiliary features.

To further bolster the exploitation of directional geometric information along the regression pathway, we design an Orientation-Aware Regression Enhancement Unit (OAREU) and embed it within the regression branch of the PCR Head. This module does not alter the output parameterization of the detection head. Instead, it augments the directional information of regression features at the current scale in the feature domain and incorporates auxiliary information from adjacent scales for adaptive fusion, thereby furnishing more stable and geometrically discriminative feature representations for rotated box regression. An overview of the OAREU structure is provided in Figure 5, and it primarily consists of four steps: directional enhancement, adjacent-scale auxiliary fusion, 1 × 1 projection, and channel recalibration.

Let the input regression feature at the *i*-th detection level be denoted as $F_{i}$. First, OAREU performs directional enhancement on the current-level feature to highlight the responses related to the target principal axis, edge orientation, and local geometric structure, thereby obtaining a basic enhanced feature. It is defined as follows:

(8) $$B_{i}=w_{i}F_{i}+(1-w_{i})\varphi_{i}(F_{i})$$

where φ(⋅) denotes the directional enhancement transformation applied to the current-level feature, which is used to mine local structural information with stronger directional sensitivity; $w_{i}$ denotes a learnable balancing coefficient used to adaptively trade off the original feature and the enhanced feature. This design avoids wholesale replacement of the original regression representation. Instead, it injects directional information that is more conducive to rotated geometric modeling while preserving the stability of the original semantic and spatial distribution.

However, relying solely on the current-scale feature for directional enhancement may still be circumscribed by the representational capacity of a single scale. Shallow features typically contain richer edge- and texture-related details but possess relatively weak semantic expressiveness; deep features exhibit stronger semantic abstraction yet may forfeit some fine-grained directional structure. To address this limitation, adjacent-scale auxiliary information is further introduced to complement the current-level regression representation. Concretely, the coarser-scale feature is first processed by a 1 × 1 convolution and an upsampling operation to generate an auxiliary feature aligned with the current scale, denoted as $F_{i+1}$; the finer-scale feature is transformed by a 3 × 3 convolution with stride 2 and an alignment operation to obtain a downsampled auxiliary feature, denoted as $F_{i-1}$. Subsequently, the direction-enhanced feature of the current level and the auxiliary features from the two adjacent scales are concatenated along the channel dimension to obtain a fused representation:

(9) $$M_{i}=Cat(B_{i},U_{i},D_{i})$$

where Cat(⋅) denotes the channel concatenation operation. Through this step, the regression feature at the current level not only retains its own direction-sensitive information but also assimilates contextual geometric complements from adjacent scales, thereby improving its comprehensive representational capacity for elongated ship structures, scale variations, and directional continuity.

Since channel concatenation inevitably brings feature dimension expansion and a certain degree of information redundancy, a 1 × 1 convolution is further employed to linearly project and compress the fused result, yielding a compact intermediate representation:

(10) $${\tilde{F}}_{i}=\psi_{i}(M_{i})$$

where $\psi_{i}(\cdot )$ denotes the 1 × 1 projection mapping. Through this process, directional and scale information from different sources is reorganized into a unified feature space, thereby reducing the redundant interference caused by direct concatenation and improving the efficiency of the subsequent regression branch in exploiting effective geometric cues.

On this basis, OAREU introduces a channel recalibration operation to adaptively reweight the projected feature and produce the final enhanced output:

(11) $${F_{i}}^{e}=CA({\tilde{F}}_{i})$$

where CA(⋅) denotes the channel recalibration function. This operation models the differential importance of distinct channels for the regression task, thereby adaptively amplifying responses more pertinent to target center localization, scale estimation, and angle prediction, while suppressing interference stemming from background clutter, noisy textures, or irrelevant scattering. The enhanced feature is ultimately fed into the subsequent regression prediction branch to support more stable learning of rotated bounding box parameters.

OAREU operates principally within the regression path. Through current-scale directional enhancement, adjacent-scale auxiliary fusion, and channel recalibration, it elevates the geometric discriminability of regression features. From an implementation standpoint, OAREU introduces only a modest number of convolution, scale alignment, and channel reweighting operations. The overall structure is lightweight and can be readily embedded into multiple levels of the regression branch without appreciably inflating the model’s computational burden. Without modifying the output format of the detection head, this module augments the perception and representational capacity of regression features with respect to directional geometric information, thereby providing feature-level support for subsequent improvements in rotated box localization accuracy.

### 3.5. Uncertainty-Aware Sample Reliability Steering (UARS)

During the training of rotated ship detection models tailored for complex SAR scenes, the positive samples identified by the sample assigner constitute a heterogeneous set, and the supervision quality within this set often exhibits marked variation. Particularly in challenging inshore backgrounds, certain positive samples manifest a characteristic mismatch: the model yields relatively high target confidence in the classification branch, whereas the geometric consistency between the predicted rotated box and the ground-truth box remains low. For brevity, we refer to such samples as high-confidence, low-consistency (HCLC) positive samples. This phenomenon indicates that, while the model has already formed a confident detection of a target’s presence at the corresponding location, the fine-grained modeling of its center, scale, and rotation angle has yet to converge to a stable state. Accordingly, the regression supervision derived from HCLC samples at the current training iteration may prove unreliable.

Such samples are highly representative of complex SAR scenes. On one hand, inshore regions are frequently afflicted by sea–land clutter, strong scattering from port infrastructure, speckle noise, and partial occlusion, conditions under which the network can still extract discriminative responses sufficient to ascertain “whether a target exists.” On the other hand, ship targets themselves typically exhibit elongated profiles, arbitrary orientations, and blurred boundaries, further compounding the difficulty of accurately fitting rotated boxes in terms of position, scale, and angle. As a result, the classification and regression branches may become desynchronized for the same sample: the former may have already converged toward stability, whereas the latter may still fluctuate considerably. If such samples continue to be treated on par with high-quality positive samples during training, unstable supervision signals will be persistently injected into the regression branch, leading to oscillatory regression loss, disordered gradient directions, and ultimately constrained localization performance under high-IoU thresholds. This effect is typically more pronounced on metrics that emphasize fine localization quality, such as AP_50:95_.

Nevertheless, directly discarding or hard-filtering such samples is equally inadvisable. High-confidence–low-consistency samples are not tantamount to erroneous samples. A substantial fraction of them remain challenging examples within the normal learning trajectory, differing only in that their geometric modeling has yet to converge at the current training stage. Indiscriminate removal might temporarily reduce noisy gradients, but it could also impair the model’s adaptability to complex backgrounds, occluded targets, and ambiguous-boundary instances. Hence, such samples are better suited to a conservative and progressive regulation strategy rather than a one-shot elimination mechanism. Motivated by this observation, we propose an Uncertainty-Aware Sample Reliability Steering (UARS) module to identify and flexibly manage the regression contribution of such potentially unreliable positive samples online during training.

**Figure 6:**
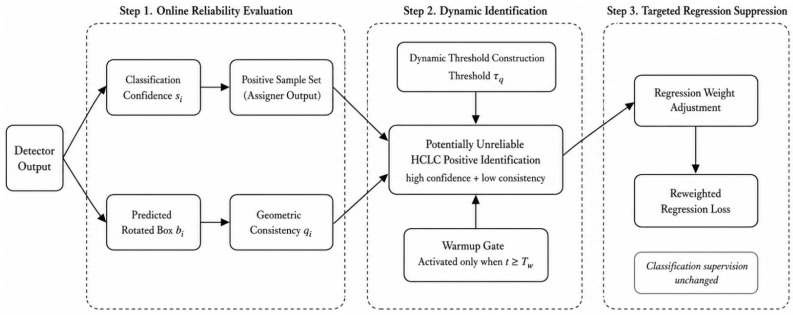
Overall workflow of the Uncertainty-Aware Sample Reliability Steering (UARS) mechanism. UARS identifies potentially unreliable HCLC positive samples through online reliability evaluation and dynamic identification, and then regulates only the regression branch while keeping classification supervision unchanged.

It is important to clarify that the term “uncertainty awareness” in UARS does not refer to explicit modeling of statistical uncertainty in the Bayesian sense, nor does it entail an additional dedicated uncertainty prediction branch. Instead, it indirectly characterizes the instability of sample supervision quality under the current training state by leveraging the discordance between classification confidence and rotated geometric consistency. In other words, UARS is not concerned with whether a sample belongs to the foreground; rather, it assesses whether the sample is suitable for participating in regression optimization with its original intensity at the current stage. Therefore, UARS neither modifies the main detector architecture nor interferes with the positive/negative sample assignment rule. Instead, it performs fine-grained reliability evaluation and training regulation within the positive sample set determined by the sample assigner. The overall workflow is depicted in Figure 6 and encompasses three primary steps: online evaluation, dynamic identification, and targeted suppression.

(1) Online Evaluation: Extracting Reliability Cues from Positive Samples

Let the positive sample set output by the sample assigner be denoted as *P*. For any positive sample i∈P, UARS extracts two complementary types of reliability cues from the classification branch and the regression branch, respectively. Specifically, the target confidence $s_{i}$ is first obtained from the classification branch to quantify the model’s confidence that a target exists at the corresponding location. Then, the geometric consistency is computed based on the rotated geometric similarity between the predicted rotated box $b_{i}$ and the ground-truth rotated box $b_{i}^{gt}$:

(12) $$q_{i}=ProbIoU(b_{i}^{gt},b_{i}),q_{i}\in [0,1]$$

where a larger $q_{i}$ indicates that the sample is better fitted in terms of position, scale, and orientation; conversely, a smaller $q_{i}$ suggests that the current regression quality remains insufficient.

It warrants emphasis that UARS does not merely reiterate the original sample assignment process. The role of the sample assigner is to determine which candidate locations are deemed positive samples, whereas UARS further evaluates whether the regression supervision of these already designated positive samples is equally reliable. The former concerns foreground/background partitioning; the latter concerns quality discrimination within the positive sample set. Precisely because their functional boundaries are clearly delineated, UARS can be seamlessly integrated into the training pipeline of existing detectors as an auxiliary module without disrupting the original sample assignment rule.

(2) Dynamic Identification: Constructing the Decision Boundary for High-Confidence–Low-Consistency Samples

After obtaining $s_{i}$ and $q_{i}$, the next step is to identify which positive samples should be regarded as potentially unreliable. Intuitively, such samples should satisfy two conditions simultaneously. First, the classification confidence is high, indicating that the model already strongly believes that a target exists at the corresponding location. Second, geometric consistency must be low, signifying that the fitting of the rotated box in terms of position, scale, and angle has yet to attain a level commensurate with the classification judgment. Only when both conditions are met does the sample exhibit the contradictory state wherein the classification response has stabilized while geometric regression still lags substantially behind.

In the decision strategy, UARS adopts a combination of a fixed classification threshold and a dynamic geometric threshold, rather than using fixed thresholds for both indicators. Specifically, the classification threshold $s_{0}$ is fixed, because the classification response usually becomes stable relatively early during training, and a fixed threshold helps maintain consistency in the decision criterion. In contrast, the geometric consistency $q_{i}$ fluctuates more significantly across different training stages, different batches, and different scene complexities. If a fixed geometric threshold were always used, normal difficult samples might be excessively suppressed in the early stage of training, whereas truly unstable samples might be difficult to identify effectively in the later stage. To address this issue, UARS adaptively constructs a dynamic threshold according to the distribution of geometric consistency among positive samples in the current batch:

(13) $$\Gamma_{q}=clip(Q_{\rho }(\{q_{i}|i\in R\}),\Gamma^{\min},\Gamma^{\max})$$

where $Q_{p}(\cdot )$ denotes the low-quantile statistic of the geometric consistency distribution of positive samples in the current batch, and $\Gamma^{\min}$ and $\Gamma^{\max}$ are used to constrain the lower and upper bounds of the threshold, so as to avoid overly high or overly low threshold values under extreme distributions. In this way, $\Gamma_{q}$ can reflect the overall geometric fitting level of the current batch while maintaining numerical stability.

Based on the above definition, the decision mask for potentially unreliable positive samples can be expressed as

(14) $$m_{i}=1(s_{i}>s_{0}\wedge u_{i}<u_{0})$$

where 1(·) denotes the indicator function, and $u_{0}$ denotes the threshold adaptively constructed from the consistency distribution of positive samples in the current batch. This decision logic precisely corresponds to the contradictory training scenario in which the model assigns high confidence to a sample at the classification level, yet the geometric fit between the rotated box and the ground-truth box remains deficient.

**Figure 7:**
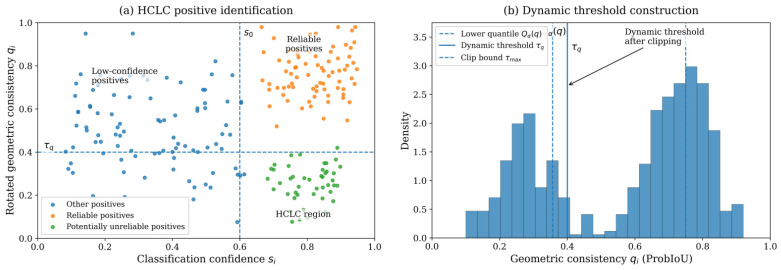
Illustration of UARS-based positive-sample reliability identification. (**a**) Potentially unreliable HCLC positives are identified using classification confidence and geometric consistency. (**b**) The dynamic threshold is constructed from the distribution of geometric consistency values with clipping bounds for stable sample regulation.

This decision logic is consistent with the schematic relationship illustrated in Figure 7. In Figure 7a, positive samples are mapped onto a two-dimensional plane of classification confidence–geometric consistency, where the horizontal axis represents the classification confidence $s_{i}$ and the vertical axis represents the geometric consistency $q_{i}$. The lower-right region partitioned by the fixed classification threshold $s_{0}$ and the dynamic geometric threshold $\Gamma_{q}$ corresponds exactly to the UARS identification region of high-confidence but low-consistency samples. The upper-right region corresponds to reliable positive samples for which both classification judgment and geometric fitting are relatively stable. By contrast, the left region usually corresponds to ordinary positive samples or normal difficult samples whose classification confidence has not yet been sufficiently established, and therefore such samples are not suitable for focused suppression at the current stage. Figure 7b further illustrates the construction of the dynamic threshold $\Gamma_{q}$: namely, a low-quantile statistic is first extracted from the ProbIoU distribution of positive samples in the current batch, and is then constrained to a preset interval through a clip operation, thereby obtaining a geometric decision boundary that can be adaptively updated with the training state.

Furthermore, UARS does not activate the above identification mechanism immediately at the onset of training. Instead, it employs a warmup gating strategy and intervenes in regression optimization only after training has entered a relatively stable phase. Let the current training epoch be denoted as *t*, and the warmup ending epoch be denoted as *t_w_*. Then, UARS starts to regulate samples according to the above rule only when *t* > *t_w_*. The reason for this design is that, in the early stage of training, the model is still in the phase of basic representation learning and preliminary geometric alignment. At that juncture, low geometric consistency is often a normative phenomenon rather than conclusive evidence of unstable supervision. Introducing suppression prematurely risks misclassifying normal difficult samples as unreliable, potentially undermining the overall convergence process.

(3) Targeted Suppression: Regression-Only Soft Downweighting

Once potentially unreliable positive samples have been identified, UARS refrains from modifying the classification loss and instead applies targeted regulation only to regression-related supervision. The rationale is that the main uncertainty of HCLC samples lies in unstable geometric optimization rather than incorrect foreground recognition. Intervening in the classification branch may disturb the relatively stable classification learning process and further increase the optimization coupling between classification and regression. Therefore, UARS strictly confines its intervention to the regression branch and acts on the supervision components that are most likely to introduce geometric learning instability:

(15) $${\bar{w}}_{i}=[(1-m_{i})+\beta m_{i}]w_{i}$$

where *β* denotes the suppression coefficient, which is used to control the attenuation strength of unreliable samples in the regression loss. When $m_{i}$ = 1, the sample is identified as a potentially unreliable sample, and its regression weight is decayed to $\beta m_{i}$; when $m_{i}$ = 0, the sample retains its unchanged original supervision strength. It can thus be seen that the essence of UARS is not to enhance high-quality samples, but rather to softly downweight high-confidence–low-consistency samples so as to reduce their disturbing effect on regression optimization.

**Figure 8:**
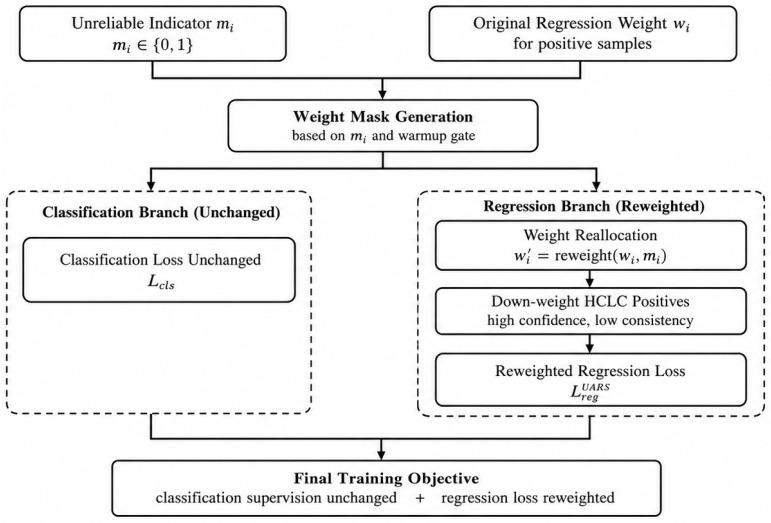
Regression-only reliability regulation mechanism in UARS. The unreliable positive indicator is used to adjust the regression weights of HCLC samples, while the classification branch remains unchanged to avoid disturbing stable classification supervision.

Moreover, to ensure that the downweighting effect is not partially nullified by the loss normalization process, UARS incorporates a critical implementation detail: although the regression weight of unreliable samples is attenuated in the numerator of the loss, the normalization denominator of the regression loss is deliberately maintained as the original sum of positive sample weights $\sum_{i}w_{i}$, rather than being synchronously replaced by the downweighted $\sum_{i}{\bar{w}}_{i}$. If both numerator and denominator were scaled down proportionally, the impact of downweighting on the final loss value and gradient update would be substantially diluted. By fixing the denominator and solely reducing the contribution of unreliable samples in the numerator, UARS faithfully transmits the design objective of “weakening unstable supervision” into the parameter update process. This is intuitively conveyed in Figure 8: the classification path preserves the original supervision unchanged, whereas the regression path adjusts sample weights according to the reliability decision results and utilizes them in the computation of regression-related losses such as Box Loss and DFL. In this way, UARS regulates only the regression contribution of HCLC samples while preserving the original classification supervision and sample assignment rule.

In summary, UARS establishes a training-level regulation mechanism that adheres to the pipeline of online evaluation → dynamic identification → targeted suppression. This method neither rewrites the original sample assignment rule, nor alters foreground/background partitioning, nor interferes with the classification learning pathway. Instead, it identifies those samples within the positive sample set for which the classification response has become relatively stable while geometric regression remains unstable, and softly suppresses their regression contribution during the middle and later stages of training, thereby mitigating the interference of unstable supervision on regression optimization.

## 4. Experiments and Analysis

### 4.1. Experimental Settings

To assess the detection performance and generalization capability of the proposed method under challenging conditions, we adopted three publicly available SAR ship detection datasets as benchmarks, as listed in Table 1: RSDD-SAR [44], SSDD+ [45], and RSAR [46]. These datasets vary in scale, scene type, target distribution, and annotation format, thereby enabling a multi-faceted evaluation of model behavior under complex backgrounds, arbitrary orientations, and multi-scale variations. Notably, RSAR was originally introduced as a multi-class SAR dataset for rotated object detection. Official statistics indicate that it contains 95,842 images and 183,534 annotated instances spanning six categories: Ship, Tank, Bridge, Aircraft, Harbor, and Car. As this study concentrates on SAR ship detection, only the Ship category annotations were preserved for training and evaluation on the RSAR dataset. According to the official category statistics, the numbers of ship instances in the training, validation, and test sets are 92,950, 10,492, and 10,700, respectively, totaling 114,142 ship instances.

Regarding data partitioning, the official splits of the public datasets were adhered to as closely as possible so as to ensure fairness and reproducibility. For RSDD-SAR and SSDD+, the publicly available test subsets were further stratified into inshore and offshore scenes in accordance with their original settings. For the RSAR dataset, the official training, validation, and test splits were adopted directly, while only the Ship annotations were retained for the single-class ship detection task. Since the official RSAR dataset lacks a demarcation between inshore and offshore scenes, only the overall test performance is reported for this dataset in the subsequent experiments. The resulting data partitioning is summarized in Table 2.

To facilitate a more comprehensive assessment of model performance on the SAR rotated ship detection task, Precision, Recall, AP_50_, and AP_50:95_ were employed as the principal evaluation metrics. Concretely, Precision and Recall quantify the accuracy of model predictions and the capacity to identify targets, respectively. AP_50_ captures the overall detection performance under a conventional IoU threshold, whereas AP_50:95_ aggregates detection results across a spectrum of IoU thresholds and thus furnishes a more faithful reflection of the model’s fine-grained localization capability for rotated bounding boxes. Given this study’s emphasis on high-quality localization of rotated targets in complex scenes, AP_50:95_ is regarded as the primary metric in the subsequent analysis.

All experiments were conducted in a Python 3.8-based deep learning environment, and model training and evaluation were primarily carried out within the Ultralytics framework. The versions of Ultralytics, PyTorch, and Torchvision utilized in the experiments were 8.3.203, 1.12.1, and 0.13.1, respectively, and the CUDA version was 11.3. To guarantee reproducibility, a unified input size and training regimen were maintained across all experiments. Unless otherwise specified, the training procedures were held constant across different comparative experiments, with the sole exception of the corresponding improved modules.

### 4.2. Module Ablation Study

To verify the independent contributions of each component and their collaborative effects, systematic ablation experiments were conducted on the three modules, namely PCR Head, RCFP, and OAREU, using YOLOv8-OBB [47] as the baseline under unified training conditions. The experiments were carried out on the official offshore and inshore test subsets of the RSDD-SAR and SSDD+ datasets. Model performance was mainly evaluated using AP_50_ and AP_50:95_. Except for the corresponding improved modules, all other training parameters and inference settings were kept unchanged. The detailed experimental settings are summarized in Table 3.

The results on the RSDD-SAR dataset are presented in Table 4. Overall, all three modules produced consistent gains when introduced individually, demonstrating the effectiveness of each design. In the offshore scene, the improvement in AP_50:95_ brought by each module was relatively limited. Specifically, PCR Head, RCFP, and OAREU improved this metric from 0.7462 for the baseline to 0.7613, 0.7613, and 0.7610, respectively. By contrast, the gains in the inshore scene were more pronounced. The baseline model achieved 0.7811 and 0.5096 in terms of AP_50_ and AP_50:95_, respectively. After introducing PCR Head, these values increased to 0.7992 and 0.5182. With RCFP, they increased to 0.7971 and 0.5159, while with OAREU, AP_50:95_ further reached 0.5269. Moreover, multi-module combinations generally yielded better overall performance. When all three modules were enabled simultaneously, the inshore AP_50_ and AP_50:95_ reached 0.8087 and 0.5353, respectively, representing improvements of 2.76 and 2.57 percentage points over the baseline. This indicates that the three modules exhibit good complementarity under complex backgrounds.

The results on the SSDD+ dataset are summarized in Table 5, and the overall trend is largely consistent with that observed on RSDD-SAR, further demonstrating the stability of the proposed method. In the offshore scene, the effects of individual modules on AP_50_ were relatively small, but stable gains were still observed on AP_50:95_. In particular, OAREU and the full three-module combination achieved 0.7954 and 0.7990, respectively, both of which were higher than the baseline value of 0.7908. The improvements in the inshore scene were more evident. After jointly enabling all three modules, AP_50_ increased from 0.8996 to 0.9188, AP_50:95_ increased from 0.6023 to 0.6364, and Precision improved from 0.8419 to 0.9067. Although slight fluctuations in Recall were observed for some module combinations, the continuous improvement in Precision and AP_50:95_ indicates that the main advantage of the proposed method lies in increasing the proportion of high-quality detections under complex background conditions.

Taken together, the results on both datasets show that PCR Head, RCFP, and OAREU provide stable gains from three different aspects, namely detection head modeling, multi-scale directional consistency, and rotated regression optimization. Among them, the improvements are more prominent in complex inshore scenes.

To further contextualize the computational complexity and practical deployment cost of the proposed forward modules, the model complexity and inference efficiency of different configurations were compared, as summarized in Table 6. All results were measured under the same input size, batch size, and hardware environment to ensure a fair comparison. The results indicate that the proposed modules introduce only a moderate additional computational burden. Among these modules, PCR Head and OAREU exert a relatively minor impact on inference speed, whereas RCFP causes a more noticeable reduction in FPS owing to its additional feature-level directional calibration operations. Although the full CORE-Net exhibits the highest computational cost among the evaluated configurations, it still maintains efficient patch-level inference capability while delivering the most favorable overall detection performance. These results demonstrate that CORE-Net achieves a reasonable trade-off between detection accuracy and deployment efficiency.

**Figure 9:**
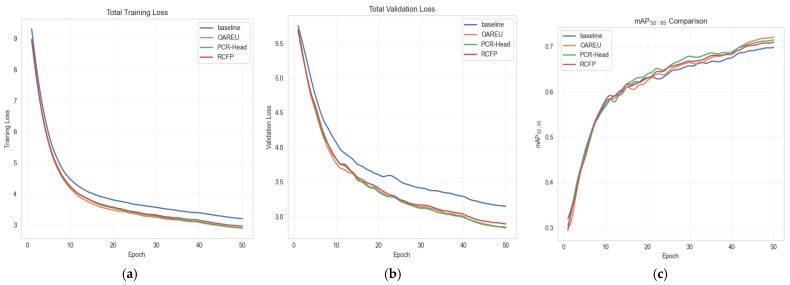
Comparison of training loss, validation loss, and AP_50:95_ curves among the baseline model, PCR Head, RCFP, and OAREU: (**a**) training loss, (**b**) validation loss, and (**c**) AP_50:95_.

On the RSDD-SAR dataset, the curves of training loss, validation loss, and AP_50:95_ for the baseline model and the models equipped with different modules are shown in Figure 9a–c. It can be observed that all models converge stably. On this basis, after introducing different modules, the fluctuations of training loss and validation loss in the middle and later stages are reduced to some extent, while the AP_50:95_ curves become smoother. This indicates that the proposed method not only improves detection performance, but also contributes to enhancing training stability.

**Figure 10:**
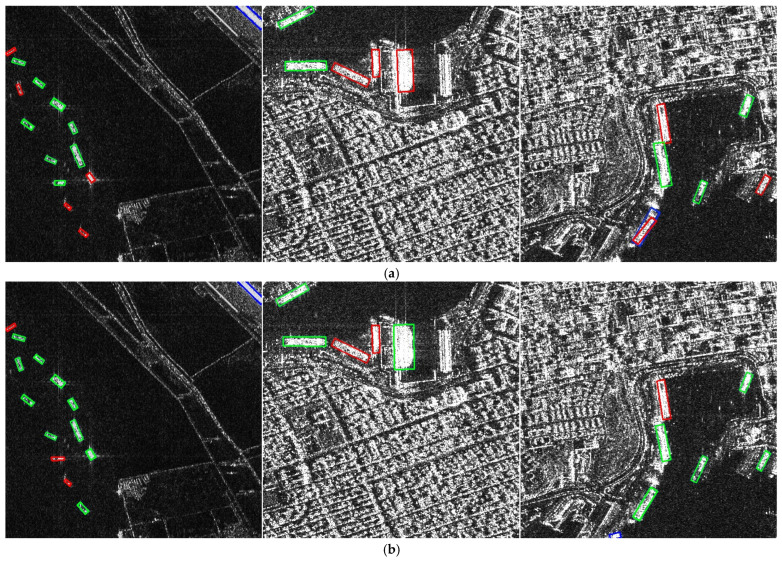

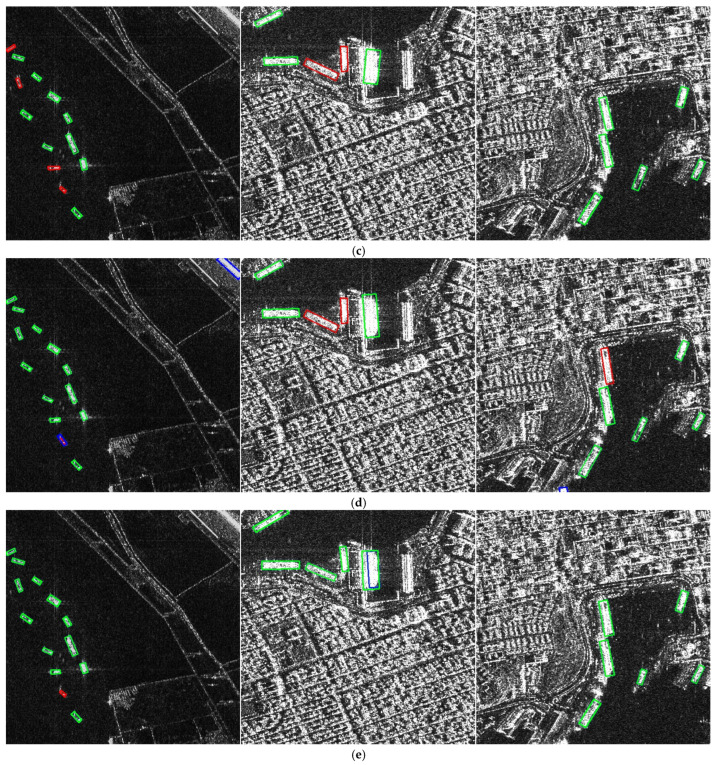
(**a**) Visual comparison of detection results under different module configurations: baseline model. (**b**) Visual comparison of detection results under different module configurations: RCFP + OAREU. (**c**) Visual comparison of detection results under different module configurations: PCR Head + RCFP. (**d**) Visual comparison of detection results under different module configurations: PCR Head + OAREU. (**e**) Visual comparison of detection results under different module configurations: PCR Head + RCFP + OAREU. Red boxes represent missed detections, blue boxes represent false alarms, and green boxes represent correctly detected targets.

A more granular visual analysis of the detection results across different module configurations is provided in Figure 10a–e, wherein red boxes denote missed detections, blue boxes signify false alarms, and green boxes correspond to correctly detected targets. Taken as a whole, relative to the baseline YOLOv8-OBB, the incorporation of different modules elevates detection quality in three representative scenarios: dense distributions, complex backgrounds, and weak targets. These improvements primarily manifest as diminished missed detections, reduced false alarms, and enhanced alignment between the rotated boxes and target contours. Specific module combinations demonstrate particular strengths in directional consistency or bounding box localization, albeit with some residual localized deficiencies. In contrast, when all three modules are engaged simultaneously, the model achieves the optimal overall balance in terms of missed detection suppression, false alarm reduction, and directional consistency of rotated boxes, further attesting to the synergistic gains realized through the three modules.

Through joint analysis of the quantitative ablation results and qualitative visualizations, it can be concluded that all three proposed modules deliver consistent performance improvements in both offshore and inshore scenes, with significantly more substantial gains in complex inshore environments. This performance difference can be attributed to the fact that inshore areas are typically characterized by stronger sea–land clutter, intense scattering from port facilities, and denser target distributions, all of which make conventional detection frameworks highly vulnerable to inconsistent directional responses and unstable regression. The proposed PCR Head, RCFP, and OAREU alleviate these core issues from complementary perspectives, thus delivering more pronounced gains in challenging inshore scenes.

### 4.3. Effectiveness Verification of UARS

To verify the independent effectiveness of UARS as a training-stage optimization strategy, as well as its generalizability across different detector architectures, UARS was integrated into both the baseline rotated detector YOLOv8-OBB and the proposed CORE-Net framework, with systematic comparative experiments conducted accordingly. The experiments primarily investigated the impact of this strategy on rotated box localization accuracy, with a specific focus on changes in AP_50:95_ under high IoU thresholds. With the exception of the integration of UARS, all other network architectures, training hyperparameters, and inference settings were kept strictly fixed. Detailed experimental settings are listed in Table 7. In addition to the general training configuration, the default hyperparameter settings of UARS are also summarized in Table 7, including the fixed classification confidence threshold, warm-up duration, quantile for dynamic geometric threshold construction, clipping range of the dynamic threshold, and default suppression coefficient. Unless otherwise specified, these hyperparameters remain fixed in all UARS-related comparative experiments. On this basis, a parameter sensitivity analysis was conducted for the core hyperparameter of UARS, the suppression coefficient *β*, using the inshore test subset of the RSDD-SAR dataset.

Unless otherwise stated, the results reported in Table 8, Table 9 and Table 10 are obtained using the default UARS settings listed in Table 7. Overall, UARS demonstrates excellent generalizability on both YOLOv8-OBB and the proposed CORE-Net, with performance gains predominantly concentrated on the AP_50:95_ metric, underscoring its particular efficacy in enhancing fine-grained rotated-box localization under high IoU thresholds.

On the RSDD-SAR dataset, both models achieved stable improvements after introducing UARS. For YOLOv8-OBB, the AP_50:95_ in the offshore scene increased from 0.7462 to 0.7549, while that in the inshore scene increased from 0.5096 to 0.5147. For CORE-Net, the offshore AP_50:95_ improved from 0.7621 to 0.7645, and the inshore AP_50:95_ improved from 0.5353 to 0.5406. Meanwhile, in the inshore scene, Precision increased from 0.8147 to 0.8297, and AP_50_ increased from 0.8087 to 0.8147. These results indicate that UARS plays a positive role in improving both localization quality and prediction accuracy in complex inshore scenes.

A similar trend can be observed on the SSDD+ dataset. For YOLOv8-OBB, after introducing UARS, the offshore and inshore AP_50:95_ values increased from 0.7908 to 0.7954 and from 0.6023 to 0.6110, respectively. For CORE-Net, the offshore AP_50:95_ improved from 0.7990 to 0.8024, while the inshore AP_50:95_ improved from 0.6364 to 0.6395. At the same time, the inshore Precision increased from 0.9067 to 0.9106. Although the inshore AP_50_ of CORE-Net changed slightly from 0.9188 to 0.9179, both AP_50:95_ and Precision were still improved, suggesting that the main benefit of UARS remains concentrated on the improvement of fine localization quality under high IoU thresholds.

The results on the RSAR dataset further verify the generalization capability of UARS. As shown in Table 10, after introducing UARS into YOLOv8-OBB, Recall, Precision, AP_50_, and AP_50:95_ increased from 0.8144, 0.8897, 0.8900, and 0.6080 to 0.8207, 0.8921, 0.8943, and 0.6109, respectively. When UARS was further combined with CORE-Net, these metrics increased from 0.8262, 0.8911, 0.9078, and 0.6114 to 0.8307, 0.8975, 0.9118, and 0.6215, respectively. Overall, UARS does not depend on a specific detector architecture and can serve as a training-stage optimization strategy to effectively suppress unreliable positive samples in different models.

Furthermore, Table 11 presents the parameter sensitivity analysis results of the suppression coefficient *β* based on the CORE-Net + UARS model on the RSDD-SAR inshore test subset. It can be observed that as *β* increases from 0.2 to 0.8, Recall shows a gradual downward trend, whereas Precision continuously increases. This indicates that different values of *β* affect the regulation strength of UARS on unreliable positive samples and further alter the trade-off between Precision and Recall. In terms of overall detection performance, when *β* = 0.6, AP_50_ reaches the highest value of 0.8147, while AP_50:95_ also attains a relatively high level of 0.5406. When *β* = 0.8, AP_50:95_ further increases to 0.5427, but Recall and AP_50_ decrease slightly. Considering detection accuracy, localization quality, and parameter stability comprehensively, *β* = 0.6 is finally selected as the default setting in this study. This outcome indicates that UARS exhibits limited sensitivity to *β*, and stable gains can be realized within a plausible value range.

**Figure 11:**
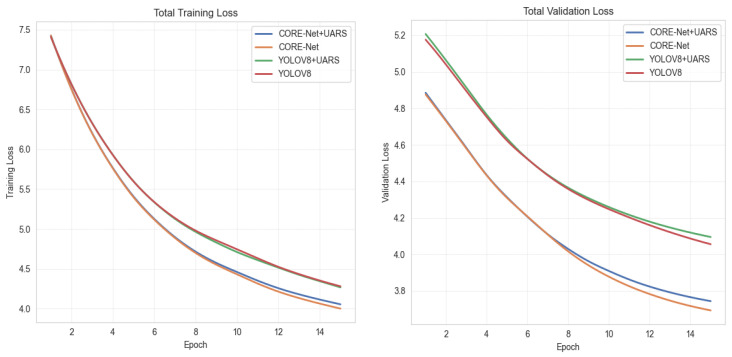
Loss curves of different models on the RSAR dataset.

As shown in Figure 11, on the RSAR dataset, the loss curves of all models decrease gradually with training epochs and stabilize in the late training stage, indicating that the overall training process of all models exhibits excellent convergence. Compared with the baseline models, both YOLOv8-OBB and CORE-Net equipped with UARS exhibit smoother loss reduction trends, with significantly reduced oscillations in the middle and late training stages. This result confirms that UARS effectively mitigates the interference of unstable supervision signals on the optimization process by suppressing the regression contribution of high-confidence, low-consistency positive samples.

**Figure 12:**
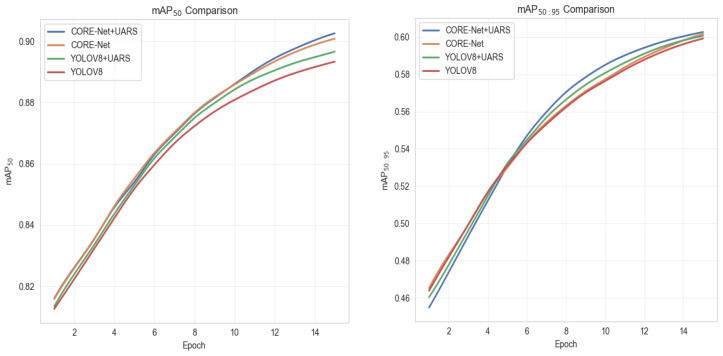
AP_50_ and AP_50:95_ curves of different models on the RSAR dataset.

As shown in Figure 12, after introducing UARS, the AP_50_ and AP_50:95_ values of the model are usually slightly lower than those of the corresponding model without UARS in the early training stage. However, as training proceeds, the models equipped with UARS gradually surpass their counterparts in the middle and later stages, and exhibit a more stable upward trend, particularly on the AP_50:95_ metric. This indicates that the main role of UARS is not to simply increase the number of detected targets, but rather to improve the rotated box localization quality under high IoU thresholds.

**Figure 13:**
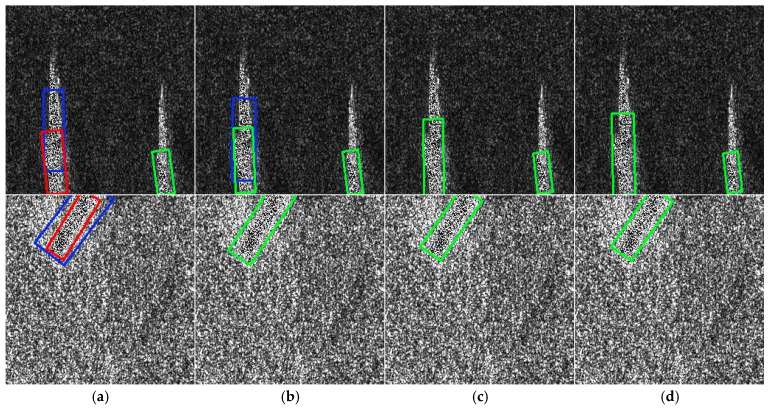
Visualization of detection results before and after applying UARS. (**a**) YOLOv8-OBB, (**b**) YOLOv8-OBB + UARS, (**c**) CORE-Net, (**d**) CORE-Net + UARS. Red boxes represent missed detections, blue boxes represent false alarms, and green boxes represent correctly detected targets.

**Figure 14:**
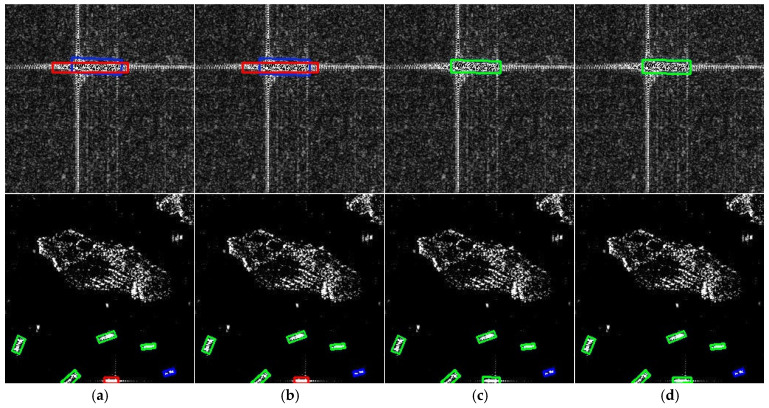
Visualization of detection results under different suppression coefficients *β*. (**a**) *β* = 0.2; (**b**) *β* = 0.4; (**c**) *β* = 0.6; (**d**) *β* = 0.8. Red boxes represent missed detections, blue boxes represent false alarms, and green boxes represent correctly detected targets.

Figure 13 presents a qualitative visual comparison of detection results before and after integrating UARS. Overall, for both YOLOv8-OBB and CORE-Net, the integration of UARS reduces false detections in complex backgrounds, improves the detection stability of weak targets and targets corrupted by local interference, and produces rotated boxes with better alignment to target contours. Figure 14 further illustrates the differences in detection results under different values of *β*. In general, different *β* values do not alter the basic detection capability of the model, but do affect false alarm suppression, missed detection control, and rotated box fitting quality to varying degrees. Among them, *β* = 0.6 achieves the optimal balance between false alarm control, missed detection suppression, and rotated box fitting quality, which is fully consistent with the quantitative results in Table 11.

In summary, as a plug-and-play training-stage optimization strategy, UARS delivers consistent and stable performance gains across different detector architectures and datasets. Its core advantages are reflected in the significant improvement of AP_50:95_ and the enhancement of training stability. When combined with the proposed feature-level and detection head-level modules, UARS can further improve the fine-grained localization capability of rotated ship detection in complex SAR scenes.

### 4.4. Comparison with Mainstream Detectors

To further validate the competitive performance detection performance of the proposed CORE-Net + UARS framework, three mainstream rotated object detectors, namely RTMDet [29], YOLOv11-OBB [48], and YOLOv12-OBB [49], were selected as comparative baselines. Experiments were conducted on the offshore and inshore test subsets of the RSDD-SAR and SSDD+ datasets. To ensure fair and rigorous comparison, all models were evaluated under the same data partition, input resolution, and basic training pipeline. With the exception of inherent differences in model architecture, all other experimental conditions were kept strictly consistent. Quantitative results of all models are reported in Table 12 and Table 13, respectively.

As can be seen from Table 12 and Table 13, the proposed CORE-Net + UARS framework maintains high detection accuracy in the offshore scenes of both datasets, and exhibits significantly more pronounced advantages in the more challenging inshore scenes. Overall, the proposed method delivers consistent and substantial improvements in AP_50:95_, demonstrating that its core benefits are reflected not only in target detection capability, but also in the significant enhancement of rotated box localization quality under high IoU thresholds.

In the offshore scene of the RSDD-SAR dataset, CORE-Net + UARS achieves Recall, Precision, AP_50_, and AP_50:95_ values of 0.9566, 0.9701, 0.9895, and 0.7645, respectively, outperforming the comparison models overall. Compared with YOLOv11-OBB, AP_50_ and AP_50:95_ are improved by 0.41 and 0.98 percentage points, respectively. Compared with YOLOv12-OBB, the corresponding improvements are 0.54 and 0.63 percentage points. These results indicate that, in offshore scenes with relatively simple backgrounds, the proposed method can still provide stable gains in high-precision rotated box fitting quality while maintaining high detection rate and prediction accuracy. In contrast, RTMDet performs markedly worse than the other OBB detectors in terms of AP_50_ and AP_50:95_ in this scene, suggesting that general-purpose detection frameworks still exhibit a certain adaptability gap for SAR rotated object detection tasks.

In the more challenging inshore scene of the RSDD-SAR dataset, the advantages of CORE-Net + UARS become more evident. Its Recall, Precision, AP_50_, and AP_50:95_ reach 0.7175, 0.8297, 0.8147, and 0.5406, respectively, all of which are the best among the compared models. Compared with YOLOv11-OBB, Recall, AP_50_, and AP_50:95_ are improved by 3.80, 5.63, and 5.83 percentage points, respectively. Compared with YOLOv12-OBB, Precision, AP_50_, and AP_50:95_ are improved by 3.48, 5.29, and 4.32 percentage points, respectively. In particular, the consistent advantage on AP_50:95_ demonstrates that the proposed method can still maintain strong regression stability and fine-grained localization capability for rotated boxes in inshore regions affected by strong sea–land clutter, scattering from port facilities, and dense target interference. On the other hand, the performance degradation of RTMDet in the inshore scene is especially significant, with AP_50:95_ reaching only 0.2842, which also indicates that complex SAR rotated ship detection imposes higher requirements on directional modeling and fine regression capability.

On the SSDD+ dataset, a trend largely consistent with that of RSDD-SAR can be observed. In the offshore scene, the Recall of CORE-Net + UARS is 0.9819, which is slightly lower than the 0.9903 of YOLOv11-OBB. However, its Precision and AP_50:95_ reach 0.9890 and 0.8024, respectively, both of which are the highest among all models. Specifically, AP_50:95_ is improved by 0.95 and 1.46 percentage points compared with YOLOv11-OBB and YOLOv12-OBB, respectively. Although its AP_50_ of 0.9930 is slightly lower than the 0.9942 of YOLOv11-OBB, the difference is only 0.12 percentage points, indicating that the proposed method still remains highly competitive overall. This suggests that, even in scenes with relatively simple backgrounds, the proposed method can further improve the high-precision fitting quality of rotated boxes while maintaining high detection accuracy.

For the inshore scene of SSDD+, the advantages of CORE-Net + UARS are also pronounced. Its Recall, Precision, AP_50_, and AP_50:95_ reach 0.8358, 0.9106, 0.9179, and 0.6395, respectively, all of which outperform the comparison models. Compared with YOLOv11-OBB, Recall, Precision, AP_50_, and AP_50:95_ are improved by 9.74, 3.17, 5.73, and 7.02 percentage points, respectively. Compared with YOLOv12-OBB, the corresponding improvements are 8.36, 5.59, 7.62, and 8.67 percentage points, respectively. These results indicate that, under complex inshore backgrounds, the proposed method exhibits stronger robustness than existing advanced OBB detectors. This further demonstrates that CORE-Net + UARS has better overall adaptability in terms of complex background modeling, multi-scale directional representation, and rotated regression optimization, and can more effectively alleviate false detections, missed detections, and inaccurate localization that commonly occur in inshore scenes.

Further comparative analysis across the two datasets reveals that our proposed CORE-Net + UARS achieves strong detection performance in offshore scenes, while delivering more substantial performance gains in highly complex inshore scenarios. In particular, its consistent performance superiority across the AP_50:95_ metric demonstrates that the core advantage of the proposed method stems primarily from its ability to improve the fine-grained localization accuracy of rotated targets, rather than only boosting detection performance under loose, conventional IoU thresholds. More precisely, the performance gains of CORE-Net + UARS are not achieved by simply increasing the number of predicted bounding boxes; instead, the method maintains superior bounding box fitting accuracy and orientation consistency under stringent high-IoU evaluation criteria.

**Figure 15:**
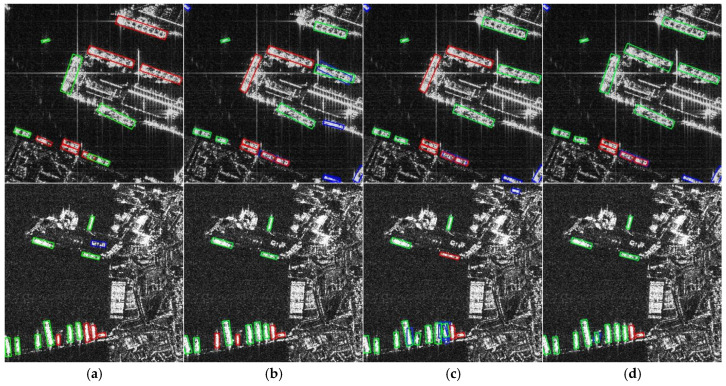
Qualitative comparison of detection results in complex SAR scenes. (**a**) RTMDet; (**b**) YOLOv11-OBB; (**c**) YOLOv12-OBB; (**d**) CORE-Net + UARS. Red boxes represent missed detections, blue boxes represent false alarms, and green boxes represent correctly detected targets.

To further visualize the performance differences across competing methods, we conducted a qualitative comparison of detection results from all evaluated detectors in complex SAR scenes, as presented in Figure 15a–d. The color coding follows the same convention as in Figure 10. Qualitative results show that RTMDet is prone to localization offsets, incomplete bounding box fitting, and false detections in scenarios with complex backgrounds and weak target responses. While YOLOv11-OBB and YOLOv12-OBB deliver relatively stable overall detection performance, they still exhibit non-negligible missed detections and orientation prediction deviations in dense inshore target clusters. In contrast, our proposed CORE-Net + UARS yields fewer false alarms in complex background regions, achieves more complete detection of weak and densely distributed targets, and generates rotated bounding boxes that align more closely with the actual contours and orientations of ship targets. These qualitative observations are generally consistent with the quantitative results presented in Table 12 and Table 13, further validating the superior robustness and adaptability of the proposed method in complex backgrounds, across multi-scale target variations, and for arbitrarily oriented targets. It should also be noted that the adopted SAR ship detection datasets provide rotated bounding-box annotations rather than pixel-level ship masks or contour annotations. Therefore, quantitative contour-based metrics cannot be reliably computed in the current benchmark setting. Instead, we use AP_50:95_ together with qualitative rotated-box alignment analysis to evaluate fine-grained localization quality.

### 4.5. Large-Scene Inference on Uncropped SAR Images

Although the quantitative experiments above are conducted on patch-based SAR ship detection datasets, practical maritime monitoring usually requires inference on large-scale SAR scenes. To further examine the applicability of CORE-Net beyond cropped image patches, we conduct additional large-scene inference experiments on uncropped SAR images. Specifically, two representative large-scale Sentinel-1 SAR scenes from LS-SSDD-v1.0 [50] and two uncropped large SAR images from RSDD-SAR are selected for qualitative validation. The selected scenes cover different imaging conditions, including open-sea backgrounds, coastal regions, island surroundings, and nearshore cluttered areas. Since public NRT/live SAR streams with complete rotated ship annotations are not available in the adopted benchmark datasets, the uncropped Sentinel-1 large-scene images are used as a practical alternative for evaluating the scalability of CORE-Net in realistic high-resolution SAR scene interpretation.

During inference, each large SAR image is divided into overlapping tiles and then fed into the trained CORE-Net model. The detected rotated bounding boxes from all image tiles are projected back to the original large-scene coordinate system. Duplicate detections in overlapping regions are further merged by non-maximum suppression. In the visualization results, green rotated bounding boxes indicate the detected ships. This sliding-window inference strategy enables CORE-Net to process high-resolution SAR scenes while preserving local ship structures and avoiding the severe target shrinkage caused by directly resizing the entire large image. The detailed settings of the large-scene inference experiments are summarized in Table 14.

**Figure 16:**
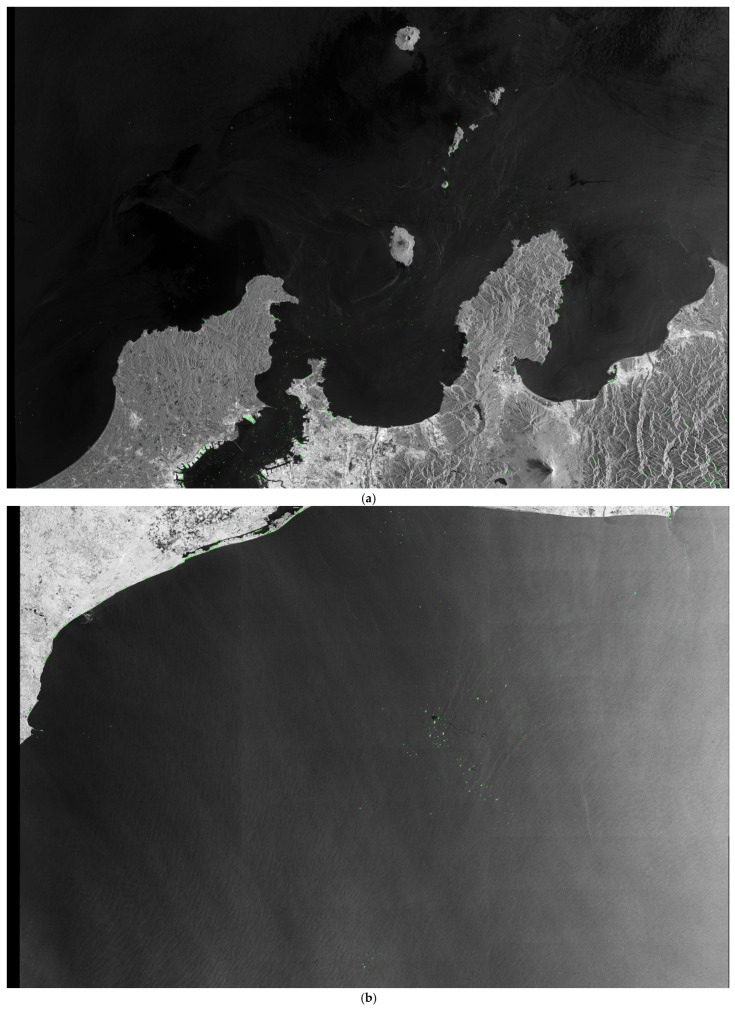

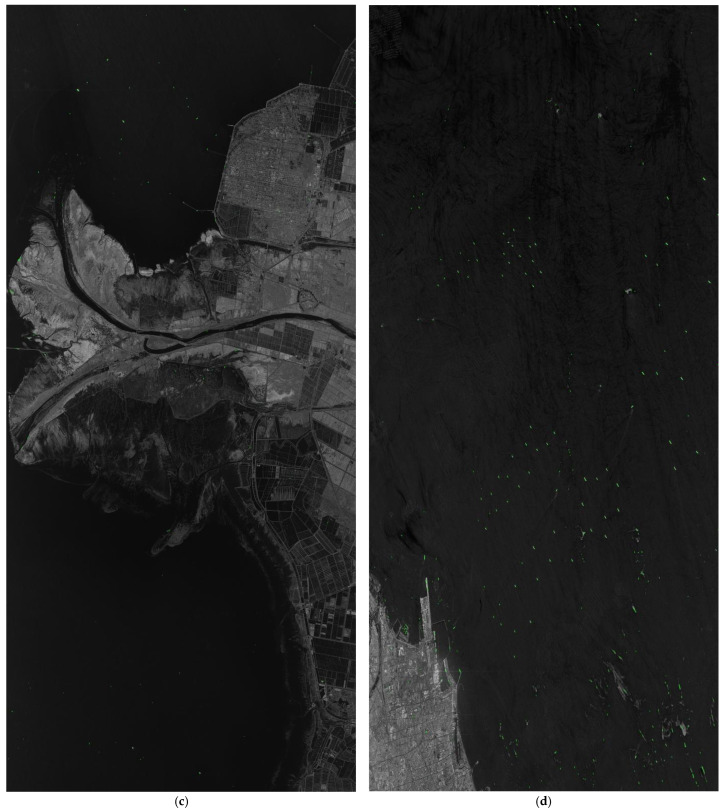
Large-scene inference results of CORE-Net on uncropped SAR images. (**a**) Sentinel-1 large-scene inference result on LS-SSDD-v1.0. (**b**) Another Sentinel-1 large-scene inference result on LS-SSDD-v1.0. (**c**) Large-scene inference result on RSDD-SAR. (**d**) Another large-scene inference result on RSDD-SAR. Green rotated bounding boxes indicate detected ships.

Figure 16 shows the large-scene inference results. Figure 16a,b are obtained from LS-SSDD-v1.0, which consists of large-scale Sentinel-1 SAR scenes. As shown in these two examples, CORE-Net can generate ship detection results in both open-sea and coastal large-scene backgrounds. This indicates that the proposed method can be applied to real Sentinel-1 large-scene SAR images rather than being limited to cropped image patches.

Figure 16c,d are obtained from the uncropped large-scene images of RSDD-SAR. The results show that CORE-Net can still produce effective detection responses under different SAR imaging sources and complex background distributions. In particular, the model can detect ships with different orientations and spatial distributions in large SAR scenes. These results further demonstrate the scalability of CORE-Net from patch-based testing to uncropped large-scene inference.

It should be noted that this experiment is mainly designed as a qualitative full-scene inference validation rather than an AP-based quantitative benchmark. This is because the large-scene images used in this section do not provide complete and unified rotated bounding-box annotations for AP_50_/AP_50:95_ evaluation. Therefore, the purpose of this experiment is to verify whether CORE-Net can effectively perform inference on real high-resolution SAR scenes. The visualization results show that CORE-Net has the potential to support large-scene SAR ship detection and practical maritime surveillance applications.

Overall, the large-scene inference results provide supplementary evidence that CORE-Net is not restricted to patch-based SAR ship detection. The model can be extended to uncropped large SAR scenes through sliding-window inference and can maintain effective ship detection responses in both Sentinel-1 and RSDD-SAR large-scene images.

## 5. Conclusions and Future Work

This study proposed CORE-Net, a collaborative optimization framework for rotated ship detection in complex SAR scenes. By integrating rotation-consistent feature pyramid calibration, progressive cascade angle regression, orientation-aware regression enhancement, and training-stage sample reliability regulation, CORE-Net jointly alleviates multiscale directional mismatch, unstable angle regression, and supervision-quality variation in positive samples. Experiments on RSDD-SAR, SSDD+, and RSAR demonstrate that the proposed method achieves consistent improvements in rotated ship detection, especially in complex inshore scenarios with dense targets, sea–land clutter, strong port scattering, and arbitrary ship orientations. The improvements in AP_50:95_ further indicate that CORE-Net enhances fine-grained rotated-box localization under stricter IoU thresholds. In addition, large-scene inference on uncropped Sentinel-1 and RSDD-SAR images shows that the proposed model can be extended from patch-based evaluation to high-resolution SAR scene interpretation through a sliding-window inference strategy.

Although CORE-Net improves rotated ship detection performance, several limitations remain and motivate future research. First, the current large-scene experiment is conducted under an offline inference setting. Since public near-real-time or live SAR streams with complete rotated ship annotations are not available in the adopted benchmark datasets, uncropped Sentinel-1 large scenes are used as a practical alternative for preliminary validation. Future work will further explore more efficient near-real-time inference strategies for wide-area maritime monitoring and investigate the operational applicability of the model in continuous SAR observation scenarios. Second, the current framework mainly operates on SAR intensity images and does not explicitly incorporate physical SAR cues, such as Doppler information, subaperture characteristics, complex-valued phase, or polarimetric features. Future studies will investigate the integration of these physically interpretable SAR cues and further explore whether CORE-Net detections can support downstream maritime monitoring tasks, such as ship motion or velocity estimation based on Doppler centroid differences and reference displacement methods. Third, the current evaluation relies on rotated bounding-box annotations. Since the adopted datasets do not provide pixel-level ship masks, quantitative contour- or shape-level metrics cannot be reliably computed. Future work will consider datasets with mask-level annotations or weakly supervised contour modeling to further evaluate the structural consistency between detected rotated boxes and actual ship contours.

## Figures and Tables

**Table 1:** Statistics of the datasets used in the experiments.

Dataset	Images	Ship Instances
RSDD-SAR	7000	10,263
SSDD+	1160	2456
RSAR	95,842	114,142

**Table 2:** Dataset splits used in the experiments.

Dataset	Training Images	Validation Images	Test Images	Scene Description
RSDD-SAR	5000	-	2000	The test set is further divided into 159 inshore and 1841 offshore images.
SSDD+	928	-	232	The test set is further divided into 46 inshore and 186 offshore images.
RSAR	78,837	8467	8538	Official split; no inshore/offshore division.

**Table 3:** Settings for the module ablation experiments of CORE-Net.

Category	Setting
Baseline Model	YOLOv8-OBB
Improved Modules	PCR Head, RCFP, OAREU
Input Size	512 × 512
Optimizer	AdamW
Initial Learning Rate	0.01
Momentum	0.937
Weight Decay	0.0005
Warm-up	3 epochs
Batch Size	4
Training Epochs	50 epochs
Data Augmentation	Mosaic, random translation, scale jittering, horizontal flipping, etc.
close_mosaic	10
Inference IoU Threshold	0.7
Experimental Datasets	RSDD-SAR, SSDD+
Test Scenes	Offshore, Inshore
Core Metrics	AP_50_, AP_50:95_

**Table 4:** Ablation results on the RSDD-SAR dataset.

PCR Head	RCFP	OAREU	Offshore Recall	Offshore Precision	Offshore AP_50_	Offshore AP_50:95_	Inshore Recall	Inshore Precision	Inshore AP_50_	Inshore AP_50:95_
			0.9528	0.9629	0.9855	0.7462	0.7198	0.7958	0.7811	0.5096
**√**			0.9573	0.9590	0.9872	0.7613	0.7215	0.7813	0.7992	0.5182
	**√**		0.9501	0.9689	0.9886	0.7613	0.6997	0.8143	0.7971	0.5159
		**√**	0.9510	0.9706	0.9882	0.7610	0.7232	0.7926	0.7968	0.5269
**√**	**√**		0.9499	0.9690	0.9875	0.7657	0.7069	0.7945	0.7879	0.5212
**√**		**√**	0.9645	0.9543	0.9877	0.7595	0.7164	0.7871	0.7965	0.5280
	**√**	**√**	0.9580	0.9635	0.9879	0.7642	0.7030	0.8140	0.7972	0.5311
**√**	**√**	**√**	0.9560	0.9675	0.9884	0.7621	0.7013	0.8147	0.8087	0.5353

Note: The symbol “√” indicates that the corresponding module is enabled.

**Table 5:** Ablation results on the SSDD+ dataset.

PCR Head	RCFP	OAREU	Offshore Recall	Offshore Precision	Offshore AP_50_	Offshore AP_50:95_	Inshore Recall	Inshore Precision	Inshore AP_50_	Inshore AP_50:95_
			0.9840	0.9801	0.9936	0.7908	0.8358	0.8419	0.8996	0.6023
**√**			0.9866	0.9857	0.9933	0.7875	0.8547	0.8352	0.9009	0.6276
	**√**		0.9759	0.9849	0.9940	0.7902	0.8323	0.8721	0.9174	0.6230
		**√**	0.9755	0.9892	0.9918	0.7954	0.8023	0.9193	0.8905	0.6230
**√**	**√**		0.9840	0.9842	0.9926	0.7851	0.8321	0.9155	0.9118	0.6225
**√**		**√**	0.9804	0.9920	0.9942	0.7859	0.8198	0.9235	0.9131	0.6317
	**√**	**√**	0.9866	0.9727	0.9934	0.7974	0.8140	0.8608	0.8987	0.6224
**√**	**√**	**√**	0.9813	0.9905	0.9930	0.7990	0.8314	0.9067	0.9188	0.6364

Note: The symbol “√” indicates that the corresponding module is enabled.

**Table 6:** Model complexity and inference efficiency comparison of different module configurations.

	Params (M)	FLOPs (G)	Peak GPU Memory (MB)	Total Inference Time (ms)	FPS
YOLOv8-OBB	3.08	8.4	152	11.7	85.47
PCR Head	3.32	9.0	158	12.4	80.65
RCFP	3.37	8.7	155	13.7	72.99
OAREU	3.71	9.1	160	12.2	82.00
CORE-Net	4.61	10.8	168	15.0	66.67

Note: Params and FLOPs were calculated with an input size of 512 × 512. Total latency denotes the sum of preprocessing, network inference, and post-processing time per image. Peak GPU memory indicates the maximum reserved CUDA memory during validation. All measurements were conducted with the same batch size and hardware environment.

**Table 7:** Experimental settings of UARS.

Category	Setting
Baseline models	YOLOv8-OBB, CORE-Net
Comparative models	YOLOv8-OBB + UARS, CORE-Net + UARS
Input size	512 × 512
Optimizer	AdamW
Initial learning rate	0.01
Momentum	0.937
Weight decay	0.0005
Warm-up	3 epochs
Batch size	4
Training epochs	RSDD-SAR/SSDD+: 50 epochs; RSAR: 15 epochs
Data augmentation	Mosaic, random translation, scale scaling, horizontal flipping, etc.
close_mosaic	10
Inference IoU threshold	0.7
Experimental datasets	RSDD-SAR, SSDD+, RSAR
Test scenes	RSDD-SAR and SSDD+: offshore and inshore; RSAR: overall test set
Core evaluation metrics	AP_50_, AP_50:95_
Fixed confidence threshold *s*_0_	0.60
Warm-up ending epoch *t_w_*	8 epochs
Quantile for dynamic threshold construction *q*	0.20
$\mathrm{Clipping}\;\mathrm{range}\;\mathrm{of}\;\mathrm{dynamic}\;\mathrm{threshold}\;\Gamma^{\min}$ $\mathrm{and}\;\Gamma^{\max}$	[0.25, 0.70]
Default suppression coefficient *β*	0.6
Application scope of UARS	Regression branch only; classification supervision remains unchanged

**Table 8:** UARS results on the RSDD-SAR dataset.

Model	Offshore Recall	Offshore Precision	Offshore AP_50_	Offshore AP_50:95_	Inshore Recall	Inshore Precision	Inshore AP_50_	Inshore AP_50:95_
YOLOv8-OBB	0.9528	0.9629	0.9855	0.7462	0.7198	0.7958	0.7811	0.5096
CORE-Net	0.9560	0.9675	0.9884	0.7621	0.7013	0.8147	0.8087	0.5353
YOLOv8-OBB + UARS	0.9555	0.9736	0.9872	0.7549	0.7245	0.8016	0.7888	0.5147
CORE-Net + UARS	0.9566	0.9701	0.9895	0.7645	0.7175	0.8297	0.8147	0.5406

**Table 9:** UARS results on the SSDD+ dataset.

Model	Offshore Recall	Offshore Precision	Offshore AP_50_	Offshore AP_50:95_	Inshore Recall	Inshore Precision	Inshore AP_50_	Inshore AP_50:95_
YOLOv8-OBB	0.9840	0.9801	0.9936	0.7908	0.8358	0.8419	0.8996	0.6023
CORE-Net	0.9813	0.9905	0.9930	0.7990	0.8314	0.9067	0.9188	0.6364
YOLOv8-OBB + UARS	0.9820	0.9847	0.9931	0.7954	0.8406	0.8411	0.9073	0.6110
CORE-Net + UARS	0.9819	0.9890	0.9930	0.8024	0.8358	0.9106	0.9179	0.6395

**Table 10:** UARS results on the RSAR dataset.

Model	Recall	Precision	AP_50_	AP_50:95_
YOLOv8-OBB	0.8144	0.8897	0.8900	0.6080
CORE-Net	0.8262	0.8911	0.9078	0.6114
YOLOv8-OBB + UARS	0.8207	0.8921	0.8943	0.6109
CORE-Net + UARS	0.8307	0.8975	0.9118	0.6215

**Table 11:** Sensitivity analysis of suppression coefficient *β* in UARS on the Inshore subset of RSDD-SAR.

*β*	Recall	Precision	AP_50_	AP_50:95_
0.2	0.7245	0.8179	0.8044	0.5312
0.4	0.7198	0.8251	0.8041	0.5367
0.6	0.7175	0.8297	0.8147	0.5406
0.8	0.7148	0.8487	0.8129	0.5427

**Table 12:** Comparison with mainstream detectors on the RSDD-SAR dataset.

Model	Offshore Recall	Offshore Precision	Offshore AP_50_	Offshore AP_50:95_	Inshore Recall	Inshore Precision	Inshore AP_50_	Inshore AP_50:95_
RTMDet	0.9312	0.9562	0.9031	0.5225	0.5843	0.8271	0.5210	0.2842
YOLOv11-OBB	0.9464	0.9610	0.9854	0.7547	0.6795	0.7627	0.7584	0.4823
YOLOv12-OBB	0.9478	0.9576	0.9841	0.7582	0.6594	0.7949	0.7618	0.4974
CORE-Net + UARS	0.9566	0.9701	0.9895	0.7645	0.7175	0.8297	0.8147	0.5406

**Table 13:** Comparison with mainstream detectors on the SSDD+ dataset.

Model	Offshore Recall	Offshore Precision	Offshore AP_50_	Offshore AP_50:95_	Inshore Recall	Inshore Precision	Inshore AP_50_	Inshore AP_50:95_
RTMDet	0.9761	0.9732	0.9080	0.5271	0.6572	0.8432	0.6143	0.2603
YOLOv11-OBB	0.9903	0.9789	0.9942	0.7929	0.7384	0.8789	0.8606	0.5693
YOLOv12-OBB	0.9840	0.9724	0.9927	0.7878	0.7522	0.8547	0.8417	0.5528
CORE-Net + UARS	0.9819	0.9890	0.9930	0.8024	0.8358	0.9106	0.9179	0.6395

**Table 14:** Settings of the large-scene inference experiments.

Dataset	Image Source	Number of Scenes	Large-Scene Size	Tile Size	Overlap
LS-SSDD-v1.0	Sentinel-1 SAR scenes	2	24,000 × 16,000	512 × 512	128
RSDD-SAR	TerraSAR-X and GF-3 SAR scenes	2	TerraSAR-X: 14,848 × 40,448; GF-3: 14,336 × 32,256	512 × 512	128

## Data Availability

The code presented in this article is publicly available at: https://github.com/yongqi011210/CORE-Net (accessed on 21 April 2026). The public datasets RSDD-SAR, SSDD+, and RSAR used in this study are available from the corresponding authors of the respective datasets, with detailed information provided in the References section.

## References

[1] Argenti F., Lapini A., Bianchi T., Alparone L. (2013). A Tutorial on Speckle Reduction in Synthetic Aperture Radar Images. IEEE Geosci. Remote Sens. Mag..

[2] Jiang Y., Li W., Liu L. (2021). R-CenterNet+: Anchor-Free Detector for Ship Detection in SAR Images. Sensors.

[3] He Y., Gao F., Wang J., Hussain A., Yang E., Zhou H. (2021). Learning Polar Encodings for Arbitrary-Oriented Ship Detection in SAR Images. IEEE J. Sel. Top. Appl. Earth Obs. Remote Sens..

[4] Zhang J., Xing M., Sun G.C., Li N. (2021). Oriented Gaussian Function-Based Box Boundary-Aware Vectors for Oriented Ship Detection in Multiresolution SAR Imagery. IEEE Trans. Geosci. Remote Sens..

[5] Xu Z., Gao R., Huang K., Xu Q. (2022). Triangle Distance IoU Loss, Attention-Weighted Feature Pyramid Network, and Rotated-SARShip Dataset for Arbitrary-Oriented SAR Ship Detection. Remote Sens..

[6] Ming Q., Miao L., Zhou Z., Yang X., Dong Y. (2022). Optimization for Arbitrary-Oriented Object Detection via Representation Invariance Loss. IEEE Geosci. Remote Sens. Lett..

[7] Zhao M., Zhang X., Kaup A. (2023). Multitask Learning for SAR Ship Detection With Gaussian-Mask Joint Segmentation. IEEE Trans. Geosci. Remote Sens..

[8] Xu X.W., Zhang X.L., Zhang T.W., Shao Z., Xu Y., Zeng T. (2023). SAR Ship Detection in Complex Scenes Based on Adaptive Anchor Assignment and IOU Supervise. J. Radars.

[9] Li C., Xu E.Z., Samat A., Liu W. (2025). SARFA-Net: Shape-Aware Label Assignment and Refined Feature Alignment for Arbitrary-Oriented Object Detection in Remote Sensing Images. IEEE J. Sel. Top. Appl. Earth Obs. Remote Sens..

[10] Zhang B., Jiang X., Zhou Y., Liu X.Z. (2022). A Three-Stage Cascade Rotating Regression Network for SAR Target Rotation Detection. Proceedings of the IEEE International Geoscience and Remote Sensing Symposium (IGARSS), Kuala Lumpur, Malaysia, 17–22 July 2022.

[11] Zhao C., Fu X.J., Dong J. (2025). CGA-Det: A CNN–GNN-Based Oriented SAR Ship Detector for Complex Scenes. IEEE Geosci. Remote Sens. Lett..

[12] Ming Q., Zhou Z., Miao L., Zhang H., Li L. (2021). Dynamic Anchor Learning for Arbitrary-Oriented Object Detection. Proc. AAAI Conf. Artif. Intell..

[13] Zhu M., Hu G., Li S., Zhou H., Wang S., Feng Z. (2022). A Novel Anchor-Free Method Based on FCOS + ATSS for Ship Detection in SAR Images. Remote Sens..

[14] Yuan B., Zhi X., Hu J., Zhang W. (2024). Boosting Point Set-Based Network with Optimal Transport Optimization for Oriented Object Detection. Remote Sens..

[15] Yang X., Yang J., Yan J., Zhang Y., Zhang T., Guo Z., Xian X., Fu K. (2019). SCRDet: Towards More Robust Detection for Small, Cluttered and Rotated Objects. Proceedings of the IEEE/CVF International Conference on Computer Vision (ICCV), Seoul, Republic of Korea, 27 October–2 November 2019.

[16] Dai J., Qi H., Xiong Y., Li Y., Zhang G., Hu H., Wei Y. (2017). Deformable Convolutional Networks. Proceedings of the IEEE International Conference on Computer Vision (ICCV), Venice, Italy, 22–29 October 2017.

[17] Ding J., Xue N., Long Y., Xia G.S., Lu Q. (2019). Learning RoI Transformer for Oriented Object Detection in Aerial Images. Proceedings of the IEEE/CVF Conference on Computer Vision and Pattern Recognition (CVPR), Long Beach, CA, USA, 15–20 June 2019.

[18] Li W., Chen Y., Hu K., Zhu J. (2022). Oriented RepPoints for Aerial Object Detection. Proceedings of the IEEE/CVF Conference on Computer Vision and Pattern Recognition (CVPR), New Orleans, LA, USA, 18–24 June 2022.

[19] Ma J., Wang Y., Zhang L., Li Q. (2026). FGAA-FPN: Foreground-Guided Angle-Aware Feature Pyramid Network for Oriented Object Detection. arXiv.

[20] Liu J., Chen S. A Feature-Alignment and Attention-Fusion Network for Arbitrary-Oriented Object Detection in Remote-Sensing Images. Proceedings of the International Conference on Advances in Computer Vision Research and Applications (ACVRA 2025).

[21] Gu C., Chen L., Gu L., Fu Y. (2026). Fourier Angle Alignment for Oriented Object Detection in Remote Sensing. Proceedings of the IEEE/CVF Conference on Computer Vision and Pattern Recognition (CVPR), Denver, CO, USA, 5–7 June 2026.

[22] Sun C., Xu Y., Wu Z., Wei Z. (2022). ReAFFPN: Rotation-Equivariant Attention Feature Fusion Pyramid Networks for Aerial Object Detection. Proceedings of the IEEE International Geoscience and Remote Sensing Symposium (IGARSS), Kuala Lumpur, Malaysia, 17–22 July 2022.

[23] Cai Z., Vasconcelos N. (2018). Cascade R-CNN: Delving into High Quality Object Detection. Proceedings of the IEEE/CVF Conference on Computer Vision and Pattern Recognition (CVPR), Salt Lake City, UT, USA, 18–23 June 2018.

[24] Cheng H., Guan N., Wu J., Miao Y., Li H. Learning High-Quality Bounding Box for Rotated Object Detection via Rotated Cascade Region Proposal Network. Proceedings of the 4th International Conference on Control, Robotics and Intelligent System.

[25] Han J., Ding J., Li J., Xia G.S. (2022). Align Deep Features for Oriented Object Detection. IEEE Trans. Geosci. Remote Sens..

[26] Xu Y., Fu M., Wang Q., Wang Y., Chen K., Xia G.S., Bai X. (2021). Gliding Vertex on the Horizontal Bounding Box for Multi-Oriented Object Detection. IEEE Trans. Pattern Anal. Mach. Intell..

[27] Xie X., Cheng G., Wang J., Yao X., Han J. (2021). Oriented R-CNN for Object Detection. Proceedings of the IEEE/CVF International Conference on Computer Vision (ICCV), Montreal, QC, Canada, 10–17 October 2021.

[28] Yang X., Yan J., Feng Z., He T. (2021). R3Det: Refined Single-Stage Detector with Feature Refinement for Rotating Object. Proc. AAAI Conf. Artif. Intell..

[29] Lyu C., Zhang W., Huang H., Zhou Y., Wang Y., Liu Y., Zhang S., Chen K. (2022). RTMDet: An Empirical Study of Designing Real-Time Object Detectors. arXiv.

[30] Crisp D.J. (2004). The State-of-the-Art in Ship Detection in Synthetic Aperture RADAR Imagery.

[31] Li J., Xu C., Su H., Gao L., Wang T. (2022). Deep Learning for SAR Ship Detection: Past, Present and Future. Remote Sens..

[32] Zhang T., Ji J., Li X., Yu W., Xiong H. (2019). Ship Detection from PolSAR Imagery Using the Complete Polarimetric Covariance Difference Matrix. IEEE Trans. Geosci. Remote Sens..

[33] Zhou H., Geng Z., Sun M., Wu L., Yan H. (2025). Context-Guided SAR Ship Detection with Prototype-Based Model Pretraining and Check–Balance-Based Decision Fusion. Sensors.

[34] Ke H., Ke X., Zhang Z., Chen X., Xu X., Zhang T. (2025). SLA-Net: A Novel Sea–Land Aware Network for Accurate SAR Ship Detection Guided by Hierarchical Attention Mechanism. Remote Sens..

[35] Ke H., Ke X., Yan Y. (2024). Laplace & LBP Feature Guided SAR Ship Detection Method with Adaptive Feature Enhancement Block. Proceedings of the 2024 IEEE 6th Advanced Information Management, Communicates, Electronic and Automation Control Conference (IMCEC), Chongqing, China, 24–26 May 2024.

[36] Shrivastava A., Gupta A., Girshick R. (2016). Training Region-Based Object Detectors with Online Hard Example Mining. Proceedings of the IEEE Conference on Computer Vision and Pattern Recognition (CVPR), Las Vegas, NV, USA, 27–30 June 2016.

[37] Lin T.Y., Goyal P., Girshick R., He K., Dollár P. (2017). Focal Loss for Dense Object Detection. Proceedings of the IEEE International Conference on Computer Vision (ICCV), Venice, Italy, 22–29 October 2017.

[38] Zhang S., Chi C., Yao Y., Lei Z., Li S.Z. (2020). Bridging the Gap Between Anchor-Based and Anchor-Free Detection via Adaptive Training Sample Selection. Proceedings of the IEEE/CVF Conference on Computer Vision and Pattern Recognition (CVPR), Seattle, WA, USA, 13–19 June 2020.

[39] Ge Z., Liu S., Wang F., Li Z., Sun J. (2021). OTA: Optimal Transport Assignment for Object Detection. Proceedings of the IEEE/CVF Conference on Computer Vision and Pattern Recognition (CVPR), Virtual, 20–25 June 2021.

[40] Jiang B., Luo R., Mao J., Xiao T., Jiang Y. Acquisition of Localization Confidence for Accurate Object Detection. Proceedings of the European Conference on Computer Vision (ECCV).

[41] Tian Z., Shen C., Chen H., He T. (2019). FCOS: Fully Convolutional One-Stage Object Detection. Proceedings of the IEEE/CVF International Conference on Computer Vision (ICCV), Seoul, Republic of Korea, 27 October–2 November 2019.

[42] Li X., Wang W., Wu L., Chen S., Hu X., Li J., Tang J., Yang J. Generalized Focal Loss: Learning Qualified and Distributed Bounding Boxes for Dense Object Detection. Proceedings of the Advances in Neural Information Processing Systems (NeurIPS).

[43] Gal Y., Ghahramani Z. Dropout as a Bayesian Approximation: Representing Model Uncertainty in Deep Learning. Proceedings of the International Conference on Machine Learning (ICML).

[44] Xu C.A., Su H., Li J.W., Liu Y., Yao L.B., Gao L., Yan W.J., Wang T.Y. (2022). RSDD-SAR: Rotated Ship Detection Dataset in SAR Images. J. Radars.

[45] Zhang T.W., Zhang X.L., Li J.W., Xu X.W., Wang B.Y., Zhan X., Xu Y.Q., Ke X., Zeng T.J., Su H. (2021). SAR Ship Detection Dataset (SSDD): Official Release and Comprehensive Data Analysis. Remote Sens..

[46] Zhang X., Yang X., Li Y., Yang J., Cheng M.-M., Li X. (2025). RSAR: Restricted State Angle Resolver and Rotated SAR Benchmark. Proceedings of the IEEE/CVF Conference on Computer Vision and Pattern Recognition (CVPR), Nashville, TN, USA, 10–17 June 2025.

[47] Jocher G., Chaurasia A., Qiu J. (2023). Ultralytics YOLO.

[48] Khanam R., Hussain M. (2024). YOLOv11: An Overview of the Key Architectural Enhancements. arXiv.

[49] Tian Y., Ye Q., Doermann D. (2025). YOLOv12: Attention-Centric Real-Time Object Detectors. arXiv.

[50] Zhang T.W., Zhang X.L., Ke X., Zhan X., Shi J., Wei S., Pan D., Li J., Su H., Zhou Y. (2020). LS-SSDD-v1.0: A deep learning dataset dedicated to small ship detection from large-scale Sentinel-1 SAR images. Remote Sens..

